# Large-scale genomic analysis places Chinese CC398 as a persistent human-associated MSSA lineage apart from the dominant global LA-MRSA clade

**DOI:** 10.1128/msystems.00621-26

**Published:** 2026-09-11

**Authors:** Guilai Jiang, Changyuan Guo, Yulin Hua, Chaoshen Wu, Shengkai Li, Yuezhuo Wang, Jun Zhao, Linli Ji, Yijie Cheng, Zhemin Zhou, Xuejie Wu, Heng Li

**Affiliations:** 1 Changshu Hospital Affiliated to Soochow University, Changshu No.1 People's Hospital, Soochow University 576492 , Changshu, Jiangsu, China; 2 Department of Microbiology, School of Basic Medical Sciences, Suzhou Medical College, Soochow University 74565 , Suzhou, Jiangsu, China; 3 The Second Affiliated Hospital of Soochow University, Cancer Institute, Suzhou Medical College, Soochow University 74565 , Suzhou, Jiangsu, China; 4 National Key Laboratory of Intelligent Tracking and Forecasting for Infectious Diseases, National Institute for Communicable Disease Control and Prevention, Chinese Center for Disease Control and Prevention 12415, Beijing, China; 5 Suzhou BenQ Medical Center, Suzhou, Jiangsu, China; School of Life Science, Hangzhou Institute for Advanced Study, Hangzhou, Zhejiang, China

**Keywords:** *Staphylococcus aureus*, core-genome multilocus sequence typing, hierarchical clustering, transnational dissemination

## Abstract

*Staphylococcus aureus* clonal complex (CC)398 has emerged as a dominant livestock-associated methicillin-resistant *S. aureus* (LA-MRSA) lineage worldwide; however, its evolutionary trajectory and regional diversification remain incompletely understood. We developed a core-genome multilocus sequence typing (cgMLST) scheme with hierarchical clustering and applied it to over 30,000 *S. aureus* genomes, revealing frequent cross-border transmission of CC398. Subsequent time-calibrated phylogenetic analysis placed the most recent common ancestor at 1942 (95% CI: 1939–1945), with the human-to-livestock host jump around 1969 (95% CI: 1968–1972). Chinese CC398 exhibits a distinct trajectory: unlike the LA-MRSA lineages dominating Europe and North America, Chinese isolates are predominantly human-associated methicillin-susceptible *S. aureus* (HA-MSSA), forming unique East Asia-specific phylogroups (SAP1, SAP2, and AP1–AP3), with distinct resistance and virulence profiles. The LA lineage remains limited in China, with multinational mixed clusters emerging only after 2019. Analysis of global transmission networks revealed a significant correlation between LA-CC398 spread and international trade in fresh swine products, while no such correlation was observed for the human-associated lineage. Beyond the established lineage markers *tet*(M) and *scn*, our analysis identified additional differentially distributed genes, including *cadC*—a chromosomal cadmium resistance regulator—as a novel HA-lineage-enriched gene whose functional role in host adaptation remains to be determined. This study reveals that CC398 followed fundamentally different evolutionary paths in China versus Western countries, challenging a one-size-fits-all model of its dissemination.

**IMPORTANCE:**

This study illustrates how large-scale microbial genomics can resolve the evolutionary origins and regional diversification of bacterial pathogens. By applying a novel cgMLST scheme to over 30,000 *S. aureus* genomes, we show that CC398 followed fundamentally different evolutionary paths in China versus Western countries—challenging the prevailing model of uniform global dissemination—and that livestock-associated MRSA expansion is closely linked to international trade in fresh pork products. These findings highlight the need for integrated surveillance across human, animal, and trade interfaces to anticipate the emergence and spread of zoonotic pathogens.

## INTRODUCTION

Methicillin-resistant *Staphylococcus aureus* (MRSA) emerged in the 1960s and expansively disseminated in the 1980s as a global concern (1, 2). The accumulation of mobile genetic elements (MGEs) that confer methicillin resistance and carry virulence factors has led to the emergence of diverse MRSA variants including hospital-acquired/human-associated MRSA (HA-MRSA), community-associated MRSA (CA-MRSA), and livestock-associated MRSA (LA-MRSA) (3, 4). The LA-MRSA, once primarily confined to agricultural settings, has now disseminated through the food chain, driving an increasing trend of reservoir colonization and infection, particularly among individuals with no direct exposure to livestock (5, 6).

The global expansion of livestock-associated *S. aureus* clonal complex (CC)398 remains a significant challenge in zoonoses, even with the ongoing surveillance of MRSA across human and veterinary health sectors (4, 7). In an earlier study, Price et al. discovered that MRSA CC398 originated from human-associated methicillin-susceptible *S. aureus* (HA-MSSA) and underwent a rapid host jump from humans to livestock (8). Recently, Fernandez and colleagues constructed a time-calibrated phylogeny to indicate the divergence event in 1974 as a key factor in the subsequent transmission of MRSA CC398 across Europe (9). However, the influence of livestock on the population dynamics of *S. aureus*, particularly the factors underlying the global dissemination of CC398 since the 1980s and its widespread circulation in swine with occasional human infections, remains poorly understood. Moreover, whether CC398 followed a uniform evolutionary trajectory across different regions remains largely unknown.

To bridge this knowledge gap, we developed a novel core-genome multilocus sequence typing (cgMLST) scheme and analyzed over 30,000 *S. aureus* genomes. Using hierarchical clustering and time-calibrated phylogeny, we characterized global CC398 population dynamics and compared them with Chinese CC398 isolates. Our results reveal that while CC398 underwent a human-to-livestock host jump around 1969 and expanded as LA-MRSA across Europe and North America, Chinese CC398 followed a markedly different path, remaining a persistent human-associated MSSA lineage. These findings challenge the prevailing model of uniform CC398 dissemination and underscore the importance of region-specific surveillance within a One Health framework.

## MATERIALS AND METHODS

### Strain collection and sequencing

A total of 898 *S. aureus* isolates were subjected to whole-genome sequencing, including 850 isolates collected by the Suzhou Center for Disease Control and Prevention (CDC) between 2009 and 2022 and 48 isolates obtained from Denmark through collaboration. All bacterial DNA was purified and extracted using the Qiagen EZ1 DNA Tissue Kit (Qiagen Sciences, Germantown, MD, USA). Genomic libraries were prepared following the Illumina standard genomic DNA library preparation protocol (using the VAHTS Universal DNA Library Prep Kit for Illumina V3), targeting an insert size of approximately 300 bp for paired-end sequencing. Sequencing was performed on the Illumina NovaSeq 6000 platform with the S4 kit (v1.5) according to the manufacturer’s instructions. Quality trimming of sequencing reads was performed using EToKi v12, followed by *de novo* assembly into contigs with SPAdes v3.13 through the EToKi assemble pipeline (10, 11).

### Screening species-representative data set

A total of 30,719 *S. aureus* genomes were downloaded from the NCBI genome database in October 2022 (Table S1) and subjected to a standardized quality-control pipeline implemented in EToKi assemble (--onlyEval). Genomes with an assembly N50 <20 kb, a total assembly size outside the range of 2.4–3.2 Mb, or more than 100 low-quality bases were excluded. After quality filtering, FastANI v1.33 was employed to calculate sequence similarity to exclude non-aureus *Staphylococcus* genomes (reference genome: GCA_000013425, threshold: 0.95) (12). Following this, kssd v1.1 (DimRdcLevel = 3) was utilized to calculate pairwise similarity between genomes for subsequent clustering (13). A single-linkage hierarchical clustering was performed using the sklearn.cluster module in Python (distance threshold: 0.0008). N50 was calculated using QUAST v5.2.0, and within each cluster, the genome with the highest N50 was selected as the representative (14). To ensure the integrity of the genome, FetchMG v1.0 (https://github.com/motu-tool/fetchMGs) was used to identify universal single-copy marker genes of these genomes (criterion: COG content > 36). Subsequent predictions included ST by MLST v2.23.0 (https://github.com/tseemann/mlst), and CC typing was determined by goeBURST v1.2.1 (15). This representative data set was derived entirely from public database genomes; the 898 newly sequenced isolates were not included at this stage.

### Establishment of cgMLST scheme and hierarchical clustering

The species-representative data set was annotated using Prokka v1.14.6 to generate GFF files for pan-genome analysis. The pan-genome was constructed using PEPPAN (https://github.com/zheminzhou/PEPPAN) with *S. aureus* reference genome GCA_000013425, and core and accessory genes were identified (16, 17). EToKi MLSTdb (minimum difference between different loci = 0.1) was used to generate exemplar alleles and construct the MLST allele database, while EToKi MLSType was used to identify the corresponding reference alleles in each genome. EToKi MLSTsum summarized the MLSType results into a whole-genome multilocus sequence typing (wgMLST) allele profile, which includes all detected loci, and generated an updated allele database. EToKi cgMLST then identified highly conserved loci from the wgMLST profile as candidates for the cgMLST scheme. Finally, MLSTsum was used again to generate the cgMLST allele profile based on the selected core loci.

The cgMLST allele profiles were used to reconstruct a Neighbor-Joining (NJ) tree using GrapeTree (https://github.com/achtman-lab/GrapeTree [18]). Hierarchical clustering was performed using HierCC, and the optimal clustering thresholds were determined using HCCeval, which selects the threshold that maximizes the silhouette coefficient—a measure of cluster cohesion ranging from −1 to 1—based on pairwise allelic distances (19).

To compare the newly developed cgMLST scheme with existing schemes, the species-representative data set was additionally typed using EToKi MLSType with the publicly available PubMLST (2,208 core genes; https://pubmlst.org/) and Ridom SeqSphere+ (1,861 core genes; https://www.cgmlst.org/ncs/) cgMLST schemes (20, 21). To evaluate the applicability of these published schemes to our data set, the core genes from each scheme were treated as candidate gene sets and processed using the same filtering workflow described above before downstream comparison.

### Robustness to sampling heterogeneity

To assess the robustness of the HC650 cluster temporal composition trends to uneven sampling across years, we performed year-stratified down-sampling on the species-representative data set. Isolates were grouped into 5-year intervals. For bins containing more than 100 strains (2005–2009, 2010–2014, 2015–2019, and 2020–2024), we randomly down-sampled to a cap of *n* = 100 strains per bin over 1,000 bootstrap iterations and recomputed the proportional composition of the major HC650 clusters. Bins with 100 or fewer strains were used at their original size. The 95% confidence intervals of the down-sampled proportions were compared against the original (full-data) proportions to evaluate stability. Chi-squared tests for trend were applied to assess temporal changes in cluster composition across bins from 2000 to 2024.

To assess the robustness of the global *S. aureus* population structure to geographic sampling bias, we performed three complementary analyses on the species-representative data set: (i) leave-one-country-out exclusion for all countries with ≥30 isolates, (ii) cumulative exclusion of the top 1, 2, 3, 5, and 10 most-represented countries, and (iii) country-stratified bootstrap resampling (1,000 iterations, resampling countries with replacement). For each analysis, HC650 cluster proportions were recomputed and compared against the full data set values.

### Antimicrobial resistance and virulence profiles

Virulence genes were identified by alignment against the Virulence Factors Database (VFDB) using BLASTp (≥90% identity, ≥60% coverage) (22). Antimicrobial resistance genes were predicted with AMRfinder v3.11.14 (≥ 90% identity, ≥50% coverage) (23). All reported values represent mean gene copy number per genome. For cross-country comparisons, the CC398 data set was dereplicated (one representative genome per hierarchical clustering unit) to avoid inflation of gene counts by clonally related strains. Dereplicated and raw distributions were compared using the Wilcoxon signed-rank test; country-level antimicrobial resistance (AMR) rankings before and after dereplication were compared using Spearman rank correlation.

### Outbreak analysis

The outbreak analysis data set comprised 358 strains, including 84 outbreak isolates sequenced in this study from the 2016 Kunshan foodborne outbreak and 274 publicly available genomes downloaded from 12 epidemiologically confirmed *S. aureus* outbreak events in five countries (NCBI BioProjects PRJEB2737, PRJEB7847, PRJNA808065, PRJNA917243, PRJNA638834, and PRJNA1048050). Of these 358 outbreak-related strains, 157 were the outbreak strains identified in the published outbreak reports (the strains involved in close transmission) and served as the benchmark set for determining the close-transmission threshold. For publicly available isolates with raw sequencing data, sequencing reads were quality-trimmed using EToKi v12 and *de novo*-assembled into contigs using SPAdes v3.13 through the EToKi assemble pipeline (10, 11). We performed genotyping of 358 outbreak-associated strains using the cgMLST scheme from this study and conducted hierarchical clustering analysis via HierCC (19). The pairwise differences between core genes for each case were calculated based on the cgMLST allele profiles to determine the preliminary threshold for close transmission (hierarchical clustering level). Then, EToKi align was used to map all genes to a reference genome (GCA_000013425), facilitating the calculation of pairwise SNP distances for each case using snp-dists (https://github.com/tseemann/snp-dists). Finally, all 30,156 public genomes together with the 898 newly sequenced isolates were typed using the novel cgMLST scheme in EToKi. The resulting allele profiles were clustered using the previously determined HierCC thresholds to identify potential global outbreak clusters. Bar plots and scatter plots were generated using the ggplot2 package in R, and phylogenetic trees were visualized using GrapeTree by the neighbor-joining method (18).

### Time-calibrated phylogenetic analysis of CC398

To maximize the representation of the CC398 lineage, an updated CC398-specific data set was compiled in July 2024. The final data set comprised 3,642 genomes, including 3,539 publicly available CC398 genomes downloaded from the NCBI and 103 CC398 isolates identified by cgMLST genotyping among the 898 newly sequenced isolates. This updated data set supplemented the public genome data set used for cgMLST scheme construction with newly available CC398 genomes and was used specifically for the CC398 phylogenetic analyses described in this section. All assembled genome sequences were aligned to the *S. aureus* ST398 reference genome of strain 71193 (GenBank accession no. CP003045.1) using EToKi align to identify core genome single-nucleotide polymorphisms (cg-SNPs) (11). Recombination events were identified and filtered using RecHMM (https://github.com/zheminzhou/redHMM). Prior to inferring the time-calibrated phylogenetic tree, temporal signals in the data were assessed using TempEst v1.5.3 (24), and genomes with anomalous signals were excluded. Subsequently, based on the 85,827 recombination-free core genome SNPs identified, a time-calibrated phylogenetic tree was constructed using TreeTime (25). The phylogenies were visualized using ITOL v6 (26). The effective population size was estimated using the Skygrowth model and visualized in R with ggplot2.

### Statistical analysis of host-switching and geographical transmission events

Host jump or geographical transmission events are identified when the parent and child nodes of a branch in a phylogenetic tree are assigned different country or host states. To quantify these events, we examined all node state information along the branches of the maximum likelihood tree. We established a set, *S*, representing all possible node states. The number of host jump or geographical transmission events was represented as *N_i_*→*j*, with *i*∈*S* and *j*∈*S* denoting the states of the parent and child nodes, respectively. Gephi v0.10.1 was used for visualizing host adaptation events.

The normalized frequency of transition from state *i* to state *j*, *T_i_*→*j*, is computed using the formula; *k*∈*S* is used to iterate and calculate the total number of transitions to state *j*.


$$T_{i\to j}=N_{i\to j}/\sum_{k\in S}N_{k\to j}$$

Furthermore, the normalized frequency of transmission from continent m to continent *n* is determined by the formula; *a* and *b* signify countries within continents m and n, respectively.


$${\overset{}{T}}_{m\to n}=\sum_{a\in m}\sum_{b\in n}T_{a\to b}$$

To evaluate the robustness of the inferred host-switching and transnational transmission patterns to sampling heterogeneity, we performed a sampling-bias robustness assessment on the time-calibrated phylogenetic tree of CC398 with ancestral state reconstructions for both country and host traits. All parent-to-child state transitions along tree branches were extracted, yielding 477 country-level and 632 host-level transitions. We assessed the correlation between per-country sampling depth (number of tips) and transition counts using Pearson correlation. To control for sampling heterogeneity, we normalized each transition count by the geometric mean of the source and target country tip counts. Per-capita transition rates (transitions per tip) were computed for each country and host category to identify signals that exceed expectation based on sampling intensity alone.

### Transmission events and the correlation between trade data

International trade data for pig-related products (2004–2020) were obtained from the Harvard Dataverse (https://dataverse.harvard.edu). Trade volumes were adjusted for inflation, expressed in USD, and aggregated. Normalized trade volumes between countries were calculated. We computed the pathogen transmission frequencies and trade frequencies between countries. Additionally, pairwise Pearson correlation coefficients (*R*) were computed to evaluate the relationships between international trade flows and between trade flows and pathogen transmission. The distances between paired countries (capitals) or other regions were obtained using the Distance Calculator (https://www.distancecalculator.net/) to evaluate the relationship between geographic distance and pathogen transmission. Linear relationship scatter plots were visualized using R with the ggplot2 package.

### Machine learning analysis of specific gene distribution in human and livestock lineages

The CC398 lineage data set was independently annotated using Prokka v1.14.6, and 8,114 pan-genes were predicted using PEPPAN (https://github.com/zheminzhou/PEPPAN) (16, 17). Given that the HA and LA lineages correspond to two deeply separated phylogenetic clades, we anticipated that the classifier would capture this population structure with high accuracy and therefore employed it primarily for feature discovery rather than prediction. The data were split into training and test sets at a 7:3 ratio, and the random forest was configured with 1,000 trees, a minimum of two samples per split, zero minimum impurity decrease, and a fixed random seed of 42. Recursive feature elimination (RFE) was used to screen for the top-contributing genes. Features (genes) were prioritized if they demonstrated differential distributions (distribution >0.8 in the human lineage and <0.15 in the livestock lineage, or >0.8 in the livestock lineage and <0.15 in the human lineage). Functional analysis of these genes was performed using the UniProt online website (https://www.uniprot.org/blast) with blastp (Target database: UniProtKB reference proteomes + SwissProt, E-Threshold = 10, Matrix = Auto-BLOSUM62, Hits = 250) to elucidate their biological significance. Receiver operating characteristic (ROC) curves were plotted using matplotlib. To distinguish whether genes identified by the random forest carry host-association signal beyond broad phylogenetic clade membership, two complementary analyses were performed. First, gene presence was stratified by host category within each lineage, and differences were assessed with Fisher’s exact test. Second, leave-one-clade-out (LOCO) validation was conducted: the classifier was retrained iteratively, each time holding out one subclade, and feature importance ranks were tracked across all folds to test whether any gene’s lineage association was driven by a single subclade.

### Statistical analysis

All statistical analyses were conducted using R v4.2.2 or Python v3.6. Pearson correlation analysis was used to assess the relationships between continuous variables. Comparisons between groups were performed using the Wilcoxon rank-sum test. *P*-values < 0.05 were considered statistically significant. For the statistical summary of host-switching and geographical transmission events, we utilized the Phylo module from the BioPython library in Python for processing. The statistical tests used for each analysis are specified in the corresponding figure legends and result descriptions.

## RESULTS

### A novel cgMLST scheme reveals the global population structure of *S. aureus*

**Fig 1:**
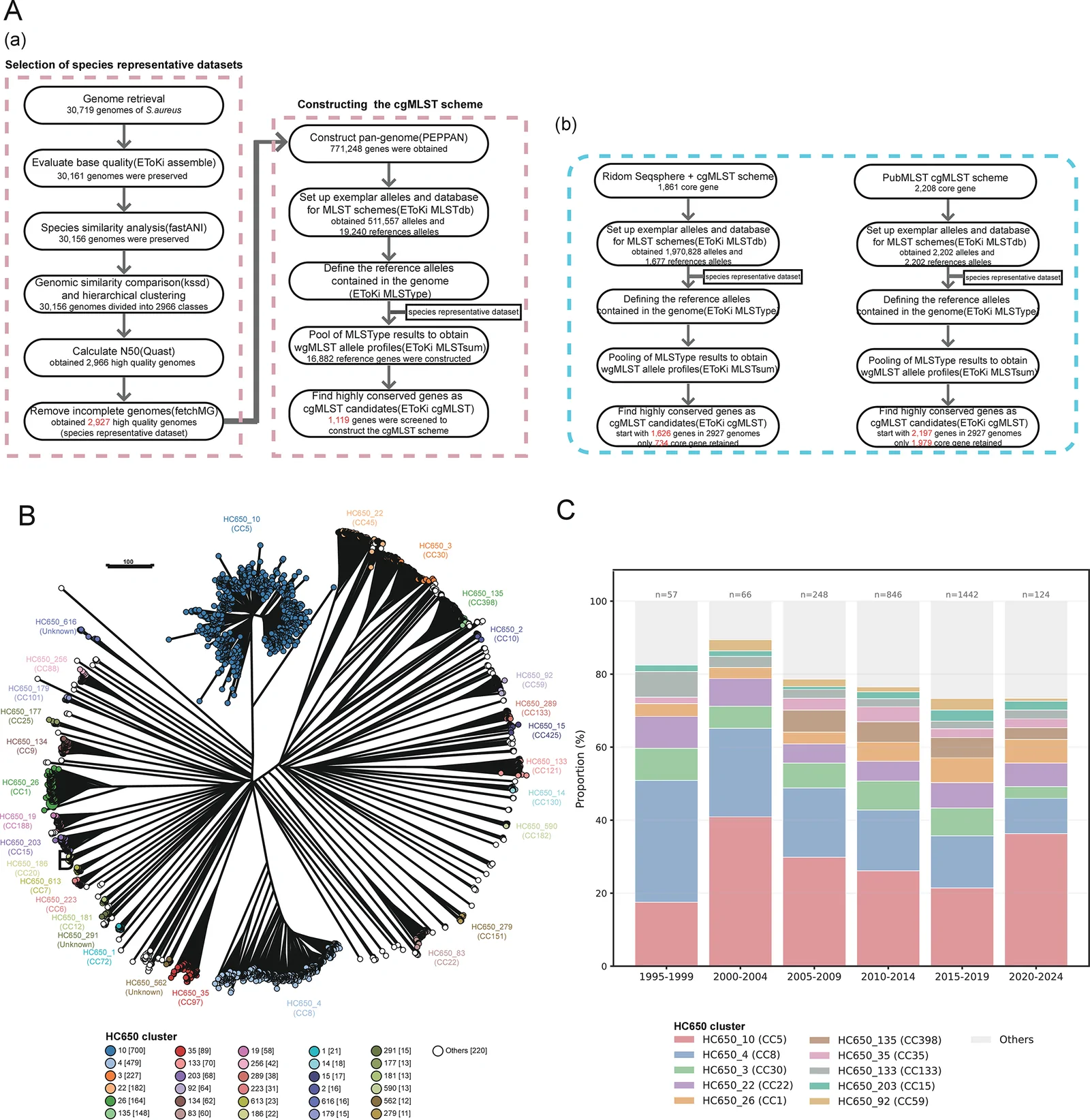
A novel cgMLST scheme for *S. aureus* reveals phylogenetic clusters and temporal dynamics. (**A**) Construction of cgMLST and HierCC clusters based on the representative *S. aureus* genomes. (**B**) Metrics comparison of the Seqsphere-cgMLST scheme and the PubMLST-cgMLST scheme. (**B**) Phylogenetic tree (RapidNJ) constructed based on 1,119 core genes from 2,927 representative genomes. Nodes are color-coded according to HC650 clusters, with the corresponding legend shown below the tree. Common HC650 clusters and their associated CCs are marked on the outer edge of the tree. (**C**) Composition of the top 10 HC650 clusters over the years.

Prior to the commencement of the project, we developed a novel core-genome MLST scheme for *S. aureus* populations (Fig. 1A, panel a). A total of 30,719 *S. aureus* genomes were enrolled for quality evaluation (accessed in October 2022). The cgMLST workflow initiated with a data set of 30,156 genomes (Table S1). After removing incomplete ones by clustering and N50 calculations, 2,927 representative genomes remained to construct the species tree (Table S2). We then obtained a pangenome comprising 771,248 genes, from which 1,119 core genes were identified as being shared by ≥95% of the representative genomes, following the procedure outlined in Materials and Methods (Fig. S1).

Two existing cgMLST schemes, PubMLST and Ridom seqsphere+, were included in our analysis process to reassess the validity and applicability of their core genes in our species-representative data set (Fig. 1A, panel b). Only 2,197 core genes out of the 2,208 from PubMLST were detected to be present in our data set as candidate genes for cgMLST. Of these, 4.51% (99/2,197), 6.05% (133/2,197), and 1.18% (26/2,197) of core genes were removed due to presence (<0.95), completeness (<0.94), and significant variation (sigma >3.0), respectively, resulting in the retention of 1,979 core genes. Only 1,626 core genes out of 1,961 core gene schemes from seqsphere were detected to be present in our data set as candidate genes for cgMLST, and almost half of them (869/1,626, 53.44%) were filtered out due to completeness (<0.94), and only 734 core genes were retained in the end (Fig. S2; Table S3). The results suggest that the core genes of the two existing cgMLST schemes may have some limitations and restrictions in our species-representative data set. Although these schemes are widely used, they showed varying degrees of challenges in the screening process of candidate genes in our study and do not fully meet our current needs for cgMLST typing.

Notably, 10.0% of strains in our species-representative data set (293/2,927) could not be confidently classified into established CC categories (Table S2); hence, we applied HierCC based on cgMLST allele profiles to provide a standardized hierarchical clustering framework for characterizing the global population structure of *S. aureus*. We assigned each cgMLST allelic profile using HierCC into a set of 1,100 hierarchical clustering levels ranging from HC0 to HC1119. Some of these clusters were found to be biologically meaningful, e.g., HC650, which corresponded to previously defined CCs (Fig. S3; Table S4).

At the HC650 resolution, the *S. aureus* CCs, namely HC650_10 (CC5) and HC650_4 (CC8), presented as the largest distinct branches within the population structure (Fig. 1B). Notably, previous studies have pinpointed CC1, CC5, CC8, CC15, and CC97 as the predominant clonal complexes (3). Analysis in 5-year intervals from 1995 to 2024 revealed marked shifts in HC650 cluster composition (Fig. 1C). Between 2000 and 2019, HC650_10 (CC5) decreased from 40.9% (2000–2004) to 21.4% (2015–2019; χ² = 30.92, *P* < 0.0001), and HC650_4 (CC8) decreased from 24.2% to 14.3% (χ² = 11.83, *P* = 0.019), with a near-perfect linear trajectory (R = −0.99, *P* = 0.002). HC650_83 (CC22) declined in parallel, from 7.6% to 4.1%. By contrast, HC650_26 (CC1) nearly doubled, rising from 3.0% (2000–2004) to 6.5% (2019–2024; R = 0.94, *P* = 0.019). HC650_135 (CC398) was absent from all 133 isolates collected before 2005, appeared at 6.0% between 2005 and 2009, and persisted without directional change through 2015–2019 (5.5%; post-emergence χ² = 1.83, *P* = 0.61). These temporal trends were robust to year-stratified down-sampling (Fig. S4; Table S5). The global HC650 cluster ranking was similarly robust to geographic sampling bias; removing the United States, which contributed 28% of the data set (820 of 2,927 isolates), shifted HC650_10 (CC5) from 23.9% to 18.4% and HC650_135 (CC398) from 5.1% to 6.6%. However, the rank order of the eight largest HC650 clusters was preserved after excluding the 10 most heavily sampled countries (Fig. S5; Table S6). Together, these analyses establish that the temporal shifts in clonal complex composition and the global ranking of major *S. aureus* lineages are not artifacts of unbalanced temporal or geographic sampling.

### Global CC398 originated from a human-to-livestock jump around 1969

**Fig 2:**
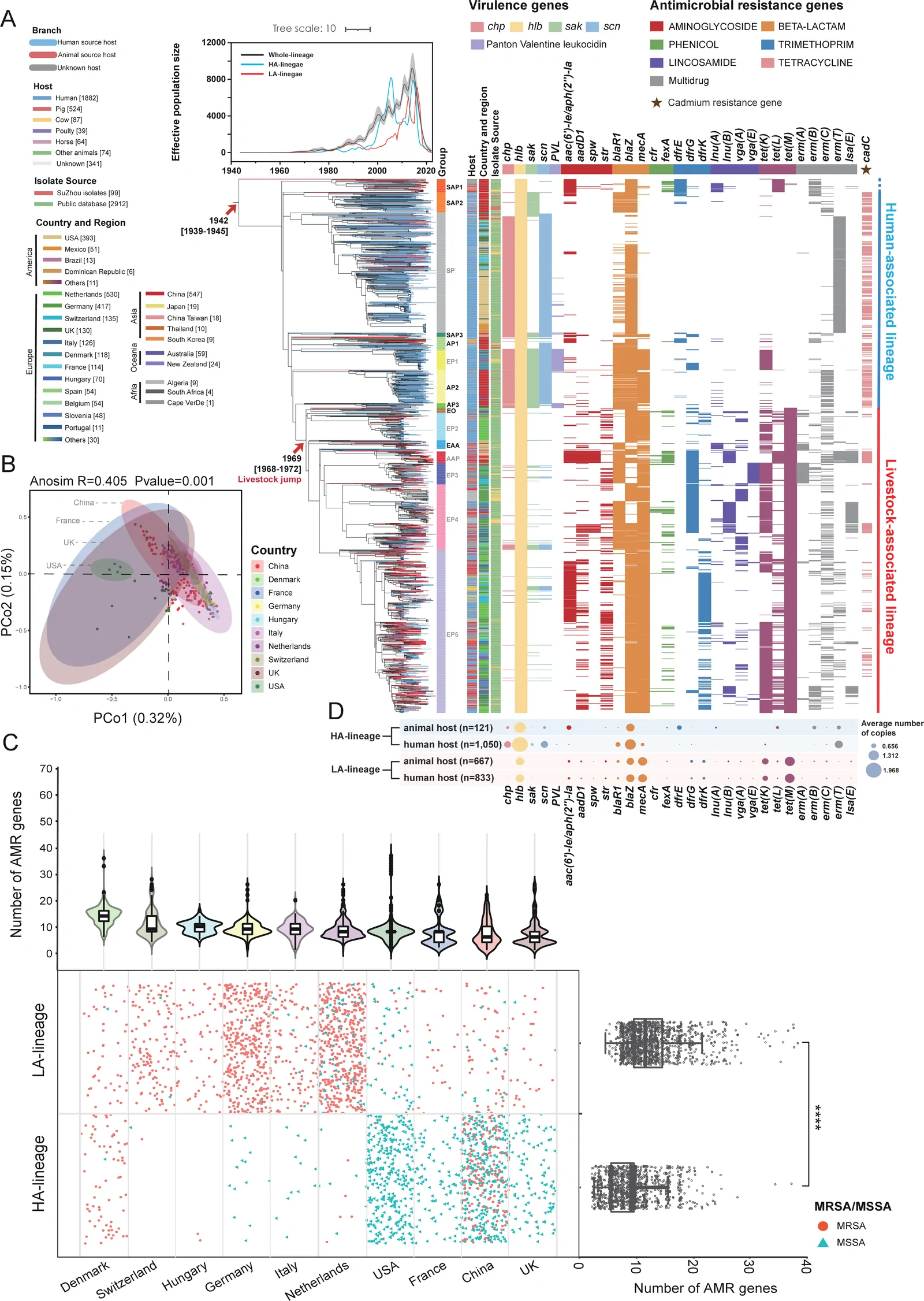
Phylogenetic tree, antimicrobial resistance, and virulence profile analysis of global HC650_135 (CC398) strains. (**A**) Time-based phylogenetic tree of global HC650_135 (CC398) strains, with analysis of isolation characteristics, antimicrobial resistance, and selected virulence profiles. The line graph shows the changes in the number of effective populations. (**B**) Principal coordinate analysis (PCoA) plot evaluating the β-diversity of antimicrobial resistance genes in strains from the top 10 countries based on Bray-Curtis distance. Statistical significance is assessed by ANOSIM similarity analysis. (**C**) Lineage distribution of MRSA/MSSA strains. The violin plot shows the mean copy number of resistance genes in strains from different countries, as well as the mean copy number of resistance genes in different lineages. (**D**) The mean copy numbers of virulence and resistance genes carried by human- and animal-derived strains within HA and LA lineages. Only isolates with available host metadata were included in this analysis.

To better understand the global distribution of *S. aureus* CC398, we gathered 3,642 isolates from this study (*n* = 103) and public databases (*n* = 3,539) to create a time-scale phylogenetic tree. Based on Treetime, we estimated the most recent common ancestor (MRCA) of CC398 originated in 1942 (95% CI 1939–1945). The switch from human to livestock hosts was estimated to have occurred around 1969 (95% CI 1968–1972), which was consistent with earlier research (Fig. 2A) (9, 27, 28).

We then compared the VFs and AMR in *S. aureus* CC398 lineages between various countries. We observed that isolates from France, the United Kingdom, and the United States shared similar AMR profiles, which were markedly distinct from those in most other European countries as assessed by principal coordinate analysis (PCoA) based on Bray-Curtis dissimilarity (ANOSIM R = 0.405, *P* = 0.001), whereas Chinese isolates showed a relatively broader range of AMR gene types (Fig. 2B). The disparities in AMR profiles among the countries corresponded to the distinct origins of the isolated bacteria, namely the variations between livestock-associated (LA) and human-associated (HA) lineages (Fig. 2C). Strains from France, the United Kingdom, and the United States were mostly part of the HA lineage, which was predominantly composed of MSSA, while strains from other European countries were mainly linked to the LA lineage, known for MRSA. The Chinese strains were largely from the HA lineage but encompassed both MSSA and MRSA. Our study also found that strains from Denmark had the highest mean number of AMR genes, with those from Switzerland, Hungary, and other nations following (*P* < 0.0001). These country-level AMR differences were not driven by the overrepresentation of outbreak-related strains (Fig. S6; Table S7).

We compared the VFs and AMR between HA and LA lineages (Table S8). Our analysis of the mean gene carriage rates showed that the LA lineage carried a broader range of antimicrobial resistance genes in both hosts. For instance, the mean copy number of *tet*(M) was 1.22 in animal-derived strains and 1.20 in human-derived strains, while *tet*(K) reached 0.81 and 0.68, respectively. In contrast, the HA lineage showed enrichment of specific genes in human-derived isolates: *erm*(*T*) was present at a mean copy number of 1.00 and *sak* at 0.39. Both were substantially lower in the LA lineage, where *erm*(*T*) and *sak* reached only 0.11 and 0.06, respectively (Fig. 2D).

To identify genomic features associated with the HA/LA divergence, we applied a Random Forest classifier to the gene presence/absence profiles of the CC398 pan-genome (8,114 genes). The model achieved high discriminatory performance (accuracy = 0.983, AUC = 0.999), consistent with the deep phylogenetic separation between the two clades (Fig. S7). Among 44 high-confidence lineage-associated genes, *tet*(M) and *scn* were the strongest discriminators—*tet*(M) was present in 99.5% of LA lineage isolates and *scn* in 95% of HA lineage isolates—consistent with their established role as lineage markers (8, 29). Notably, *cadC*, a chromosomal cadmium resistance regulator, was identified among the 27 HA-lineage-enriched genes (Fig. 2A; Table S9). Within-lineage host-stratified analysis confirmed that *cadC* is not merely a marker of HA clade membership: it was present in 75.4% of human-derived HA isolates versus 37.8% of animal-derived HA isolates (OR = 5.05, *P* < 0.0001), while remaining uniformly low across the LA lineage regardless of host. Leave-one-clade-out validation confirmed that this signal is not driven by any single subclade. These phylogeny-aware analyses indicate that *cadC* carries host-association signal beyond broad clade membership (Fig. S8 to S10; Table S10). Unlike *tet*(M) and *scn*, whose roles in CC398 host adaptation are supported by over a decade of epidemiological and genomic evidence, *cadC* represents a novel finding whose contribution to host adaptation remains to be experimentally determined.

### Chinese CC398 stands apart as a persistent human-associated MSSA lineage

**Fig 3:**
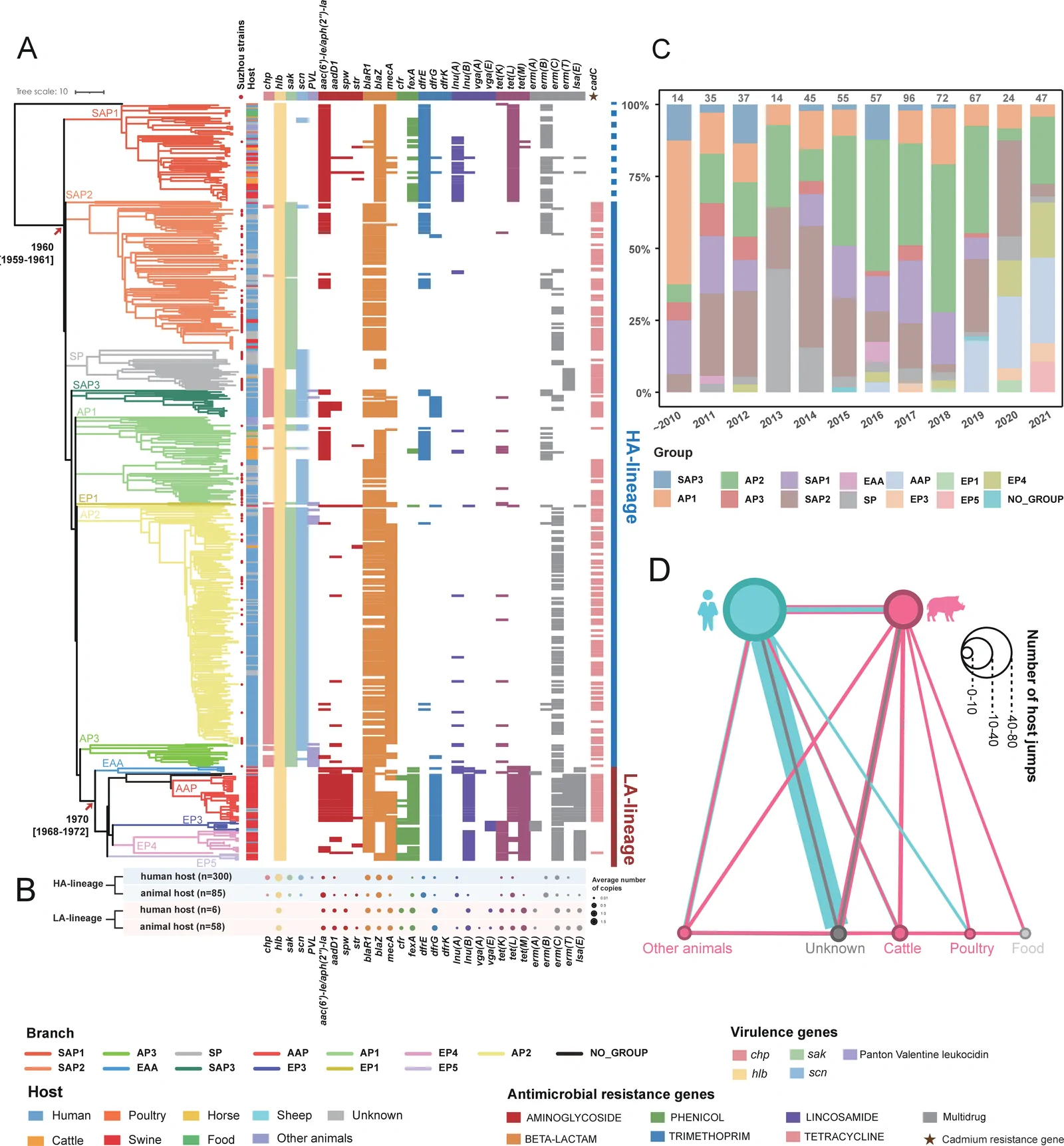
Phylogenetic tree, antimicrobial resistance, and virulence profile analysis of Chinese HC650_135 (CC398) strains. (**A**) Time-scaled phylogenetic tree of CC398 strains in China, including host origins, partial antimicrobial resistance, and virulence characteristics. (**B**) The mean copy numbers of virulence and resistance genes in human- and animal-derived strains from HA and LA lineages in China. Only isolates with available host metadata were included in this analysis. (**C**) Composition of phylogroups/sub-lineages of CC398 in China across different years. (**D**) Host-jumping frequency of the CC398 lineage. Red lines (dots) represent transmission events originating from animals, blue lines (dots) indicate transmission events originating from humans, and gray lines (dots) denote transmissions from food or unknown sources. The thickness of the lines represents the frequency of host-jumping events, and the size of the nodes reflects the total frequency of host jumping.

A time-calibrated phylogenetic tree was created to better understand the distribution of LA-*S. aureus* CC398 in East Asia (Fig. 3A). This phylogenetic analysis revealed the presence of six major clades, including Special Asian Phylogroups 1 and 2 (SAP1 and SAP2), Sensitive Phylogroup (SP), and Asian Phylogroups 1–3 (AP1–AP3). Additionally, several sub-lineages were identified, such as Special Asian Phylogroup 3 (SAP3), European Phylogroups (EP1, EP3, EP4, and EP5), European-American-Asian (EAA), and Asian-American (AAP) phylogroups. SP, AP, EP1–EP5, and AAP follow the nomenclature of Fernandez et al. (9); SAP1–SAP3, AP1–AP3, and EAA are newly defined here. When considering all Chinese CC398 strains, they largely fall within the HA lineage, with only occasional LA lineage strains observed.

Clade SAP1 (*n* = 75) was a unique MSSA lineage that emerged around 1960. This clade SAP1, which deviated from the typical characteristics of a human-associated lineage, harbored genes such as *dfrE*, *tet*(L), and *ermB* indicative of high multidrug resistance but lacked typical immune evasion cluster (IEC) genes like *chp*, *sak*, and *scn*, suggesting that it might not be primarily adapted to human hosts.

Clade SAP2 was primarily composed of human-originated MSSA. Compared with SAP1, this cluster carried *sak*, which has been associated with enhanced dissemination of *S. aureus* within the host and may facilitate infection. In contrast, the antimicrobial resistance genes *tetL* and *lnuA* were less prevalent, and only 21% (23/109) of isolates carried *dfrE* and *ermB*. Additionally, this cluster did not commonly carry the *scn* gene, which differs from the typical characteristics reported for many human-associated *S. aureus* lineages.

Clades SP and AP were previously described by Fernandez et al. (9). In the global data set (Fig. 2A), SP isolates carried *erm(T)* (92%) and the IEC genes *chp* and *scn* (95%), features of a human-adapted clade. Only 29 of 679 SP isolates originated from China; within this small subset, *scn* was universally present, but *chp* and *erm(T*) carriage was reduced (52% each). We further divided the AP cluster into SAP3, AP1, AP2, and AP3. SAP3 emerged as a highly distinctive cluster, exclusively originating from Taiwan and forming a unique group. Classified as MRSA, this cluster (*n* = 13) carried genes such as IEC, *aac(6')-le*, *blaZ*, *mecA*, *dfrG*, and *erm(B/C*). AP1 remained within the MSSA category but had lost the *chp* gene compared to SP; additionally, 27% (17/64) strains in AP1, originating from cattle, lacked the *scn* gene but carried antimicrobial resistance genes such as *aac(6')-le*, *dfrE*, and *erm(B*). However, AP2 carried *chp–sak–scn*. Together, AP2 and SAP2 comprised the two predominant HA lineage clades among Chinese CC398 isolates, with SAP2 being particularly common among the Suzhou isolates generated in this study. AP3 (*n* = 15) carried an additional Panton-Valentine leukocidin (PVL) cytotoxin. This toxin was encoded by two adjacent genes, *luk*S-PV and *luk*F-PV, located within a prophage on the bacterial chromosome, suggesting that this cluster might have evolved into a new branch following the invasion of phages.

The final major branch was the LA lineage (Fig. 3B, *n* = 70), predominantly originating from pigs and harboring a substantial number of resistance genes for aminoglycosides (*n* = 45), β-lactams (*n* = 70), phenicol (*n* = 60), trimethoprim (*n* = 64), lincosamides (*n* = 65), and tetracyclines (*n* = 70). In contrast to the evolution of the Chinese HA lineage, the LA strains did not form an independent branch in China. Instead, they formed a multinational mixed branch with strains from other countries, including EAA (*n* = 5, Europe-America-Asia), AAP (*n* = 34, America-Asia), and EP (*n* = 29, Europe). This indicated that the LA lineage exhibited cross-transmission and was present in pigs across continents simultaneously.

We compared the proportion of CC398 in China from 2010 to 2021 and found that SAP1 gradually disappeared by 2019, replaced by an increase in AAP and EP3, EP4, and EP5 within the LA lineage (Fig. 3C). Finally, we analyzed the number of host jumps of CC398 strains in China and found that jumps between humans and pigs were the most frequent, suggesting that pigs may be the primary reason for the rapid increase of CC398 in East Asia after 2019 (Fig. 3D).

### Close transmission events reveal multinational spread of CC398 between pigs and humans

**Fig 4:**
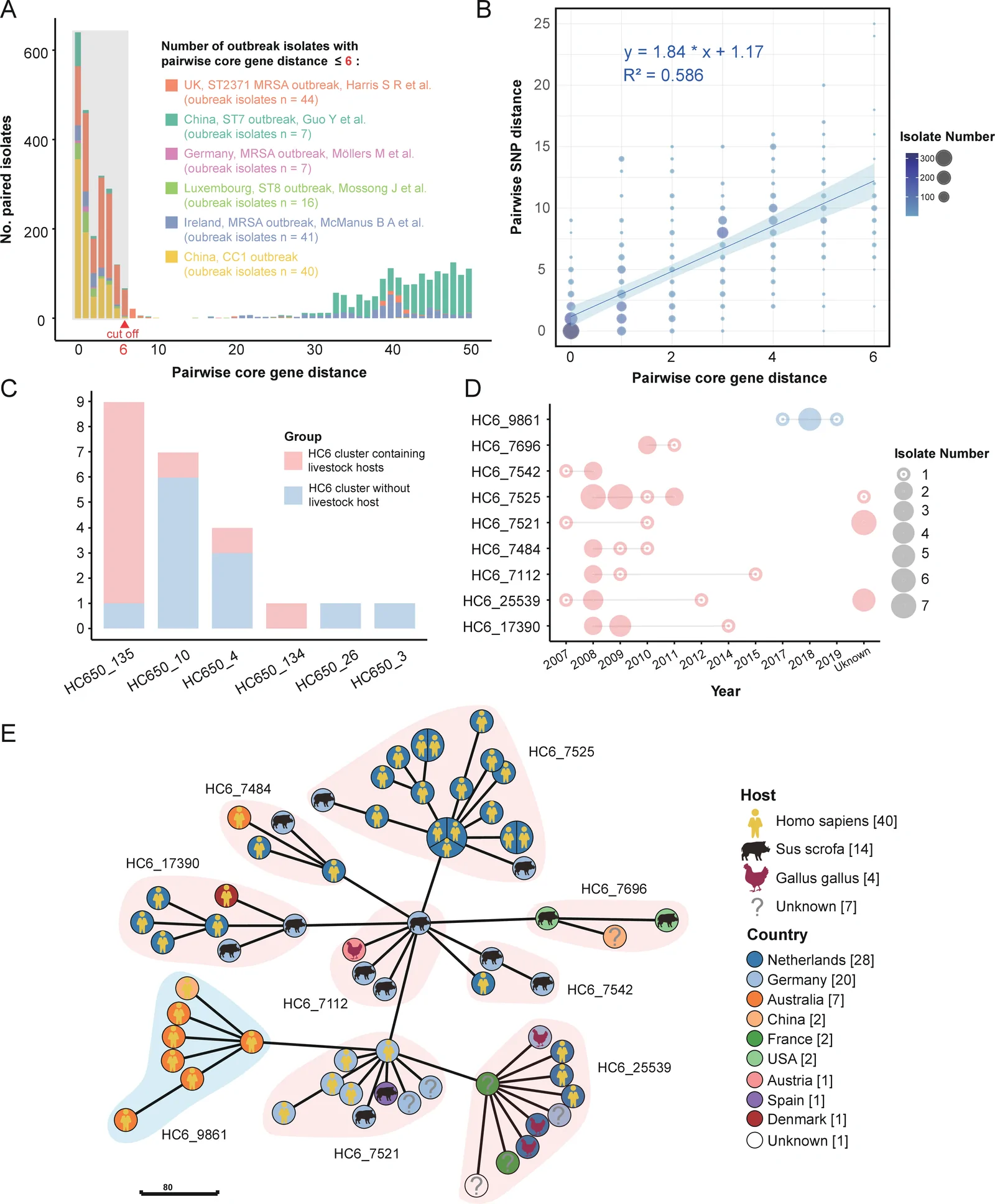
Core gene distances, lineage sources of closely transmitted clusters, and their evolutionary relationship analysis based on time and host origin in *S. aureus*. (**A**) Distribution of paired core gene distances for 358 strains from 12 cases (including seven outbreak cases from an MRSA outbreak in Ireland) and the cutoff threshold for core gene distance in closely transmitted strains. (**B**) Scatter plot showing the linear correlation between paired core gene distances and paired SNP distances in closely transmitted strains. The number of paired outbreak strains is represented proportionally by the size and color of the circles. (**C**) Composition of 23 international HC6 closely transmitted clusters, with the horizontal axis representing the corresponding HC650 clusters and the vertical axis indicating the number of strains. (**D**) Timeline of 9 HC650_135 (CC398)-related HC6 closely transmitted clusters. (**E**) Phylogenetic tree illustrating the close cross-national transmission relationship between strains. The unique 8 HC650_135 (CC398)-related HC6 closely transmitted clusters are outlined in gray. Each circle represents one or more strains, with the circle’s fill color indicating the country of origin, and the pattern inside the circle indicating the host source.

To define a benchmark for the core genes associated with close transmission events between pigs and humans, we examined 12 epidemiologically confirmed outbreaks across five countries and assessed the pairwise core gene distances of 358 strains using hierarchical clustering (Table S11). The distribution of pairwise core gene distances in the entire dataset was discontinuous: among the 358 outbreak-related strains, 157 were the outbreak strains identified in the published outbreak reports, and 155 of these 157 (98.72%) showed a core gene distance of ≤6 (Fig. 4A). A significant increase in the number of strain pairs was observed once the core gene distance surpassed 6, suggesting the involvement of strains unrelated to specific epidemiological incidents (non-outbreak strains). We validated this threshold by determining the pairwise core gene SNP distances in 155 closely transmitted strains, showing a strong correlation to the hierarchical clustering approach (R^2^ = 0.586, Fig. 4B). Consequently, we propose that a core gene distance of HC6 (a cluster with up to six allelic differences) could serve as the benchmark for genomic analysis of *S. aureus* strains involved in close or cryptic transmission events.

We endeavored to classify the global data set of *S. aureus* using the HC6 clustering approach (Table S12). At the HC6 level, a total of 1,656 HC6 clusters were identified. After excluding clusters containing laboratory strains, 23 of the 32 HC6 clusters that contained isolates from multiple countries were retained for subsequent analysis. HC650_135 (CC398) was identified in 9 out of 23 HC6 clusters as the most common clonal complex linked to close transmission events (Fig. 4C; Table S13). We then matched the metadata associated with these clusters. These HC6 clusters were found to be present in multiple countries and persisted between 2007 and 2019. Apart from HC6_9861, which had no association with livestock hosts, the remaining eight clusters were all connected to animal reservoirs (Fig. 4D).

In addition, phylogenetic analysis of the HC6 clusters provided evidence for the inter-host and cross-border dissemination of CC398. Specifically, clusters HC6_7525 (*n* = 18), HC6_7542 (*n* = 3), HC6_7484 (*n* = 4), and HC6_17390 (*n* = 7) revealed close genetic relationships between strains isolated from pigs and humans across Germany, the Netherlands, and Denmark (Fig. 4E). For example, HC6_7525 is a large, multi-country cluster comprising 16 human isolates and 2 pig isolates from the Netherlands and Germany. Similarly, HC6_17390 contains a mixture of four human and two pig isolates from Denmark, the Netherlands, and Germany. Evidence of cross-host transmission was also observed in HC6_7521 (*n* = 9), which consists of five human and two pig isolates from Germany and Spain, indicating a clear genetic link between Spanish pigs and German humans.

### Cross-border transmission of LA-CC398 and the role of pigs as a global reservoir

**Fig 5:**
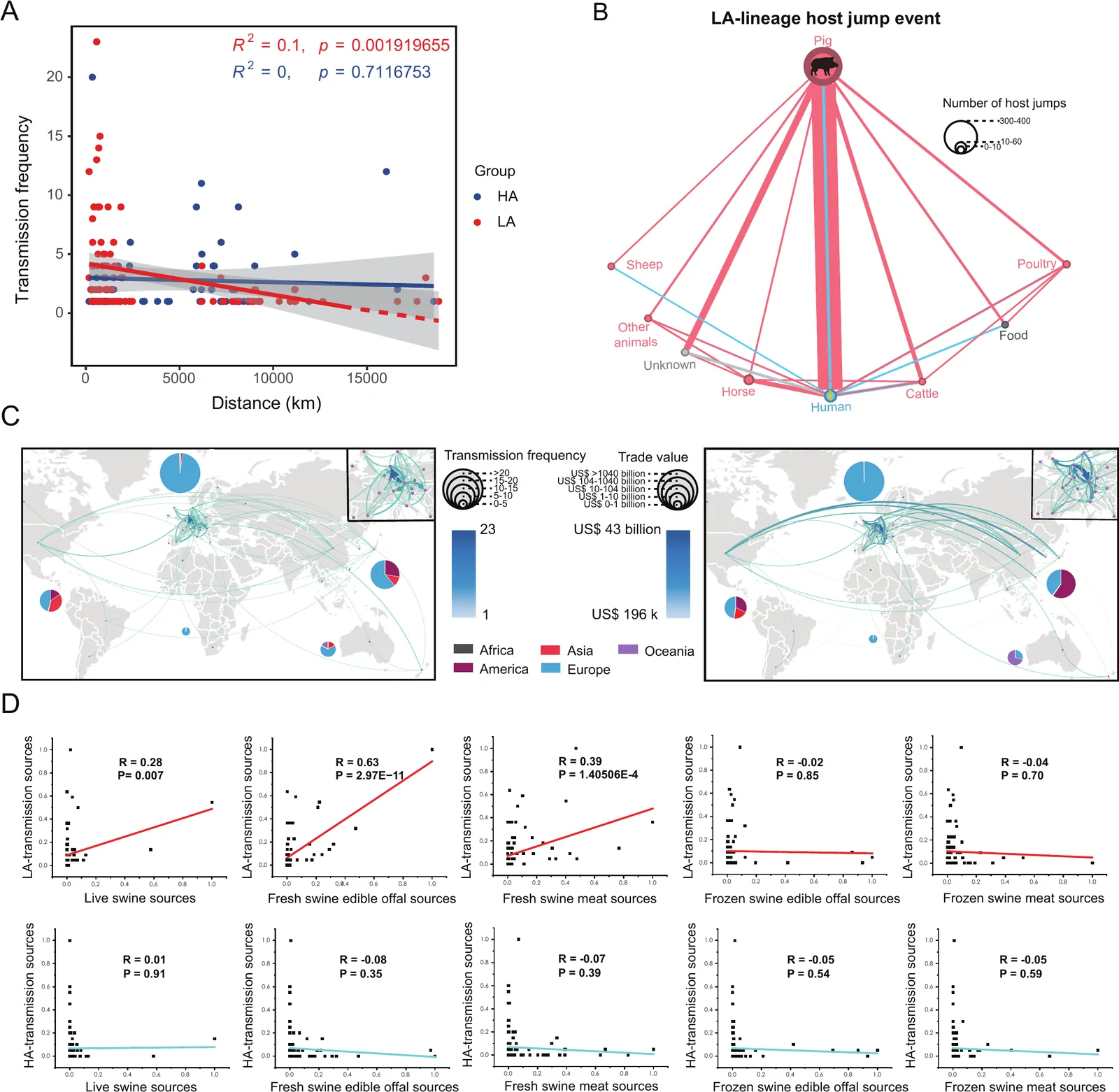
Transmission patterns of HC650_135 (CC398) livestock-associated lineages. (**A**) The linear regression of the distance (km) between countries or regions and the frequency of strain transmission. Red represents the LA lineage, and blue represents the HA lineage. (**B**) Host-jumping frequency of LA lineages. Red lines (dots) indicate transmission events originating from animal hosts, blue lines (dots) indicate transmission events originating from human hosts, and gray lines (dots) indicate transmission events originating from food or unknown hosts. Line thickness is proportional to the normalized frequency of host jump events. (**C**) Left panel: phylogeny-based transmission graph of the HC650_135 (CC398) livestock-associated lineage, inferred from the time-calibrated phylogeny. Right panel: international trade of all livestock-related products from the Harvard database. The pie chart shows the relative proportion of pathogen or product source countries compared to the target countries. Arrow colors indicate the frequency of pathogen transmission or product trade volume. (**D**) Linear regression between different categories of pork-related products (*x*-axis) and the transmission of livestock-associated lineages (red) and human-associated lineages (blue) across countries (*y*-axis). The correlation coefficient for the linear regression is also displayed on the plot.

Given that 88.9% (8/9) of the clusters from our previous analysis were associated with animal hosts, understanding the dynamics of the LA lineage is essential for managing its spread and reducing its public health threat. After reconstructing the international transmission routes of CC398 within the LA lineage, we observed a significant correlation between geographic distance (km) and cross-border transmission frequency, with closer proximity associated with higher transmission rates (*R*² = 0.1, *P* = 0.0019) (Fig. 5A). Network analysis further indicated that pigs were the sole species facilitating transmission to all other hosts, with pig-driven host jumps accounting for 75% (320/428) of all LA lineage host jump events (Fig. 5B). Additionally, 84% (242/287) of these transmissions were within Europe, consistent with livestock trade patterns from the Harvard database (Fig. 5C; Table S14). These host jump and transmission patterns were robust to sampling heterogeneity. Swine remained the predominant source in 63.3% of all host transitions after normalizing for the 3.6-fold excess of human tips over swine tips (1,883 versus 524), and the per-tip outgoing rate for swine (0.76, defined as the number of inferred host transitions originating from swine divided by the total number of swine tips in the tree) exceeded that of human (0.09), horse (0.52), and cattle (0.07). After normalizing country-level transition counts by sampling depth, the European hub structure (Germany, the Netherlands, and France as dominant sources) persisted, and the observed trade-transmission correlations were not driven by a small number of heavily sampled countries (Fig. S14; Tables S15 to S18).

Subsequently, we determined the Pearson correlation coefficients to assess the relationship between the transmission of the LA-CC398 lineage and pig trade (Fig. 5D; Table S19). Our analysis revealed a significant positive association between the dissemination of LA-CC398 and the trade volume of fresh swine offal (*R* = 0.63, *P* < 0.001), followed by fresh swine meat (*R* = 0.39, *P* < 0.001) and live swine (*R* = 0.28, *P* = 0.007), whereas no significant association was detected with frozen swine products (*R* < 0.01, *P* > 0.05). Notably, when the same analysis was applied to the HA CC398 lineage, no meaningful correlations were observed (*R* < 0.01, *P* > 0.05). These findings suggest that the global expansion of LA-CC398 is closely aligned with specific pork product trade routes, particularly fresh offal and fresh meat. This pattern underscores the potential role of trade-linked reservoirs in the international spread of livestock-derived pathogens and highlights the importance of stringent sanitary protocols during the transit of fresh animal products.

### A tripartite evolutionary model tracing the emergence of *S. aureus* CC398

**Fig 6:**
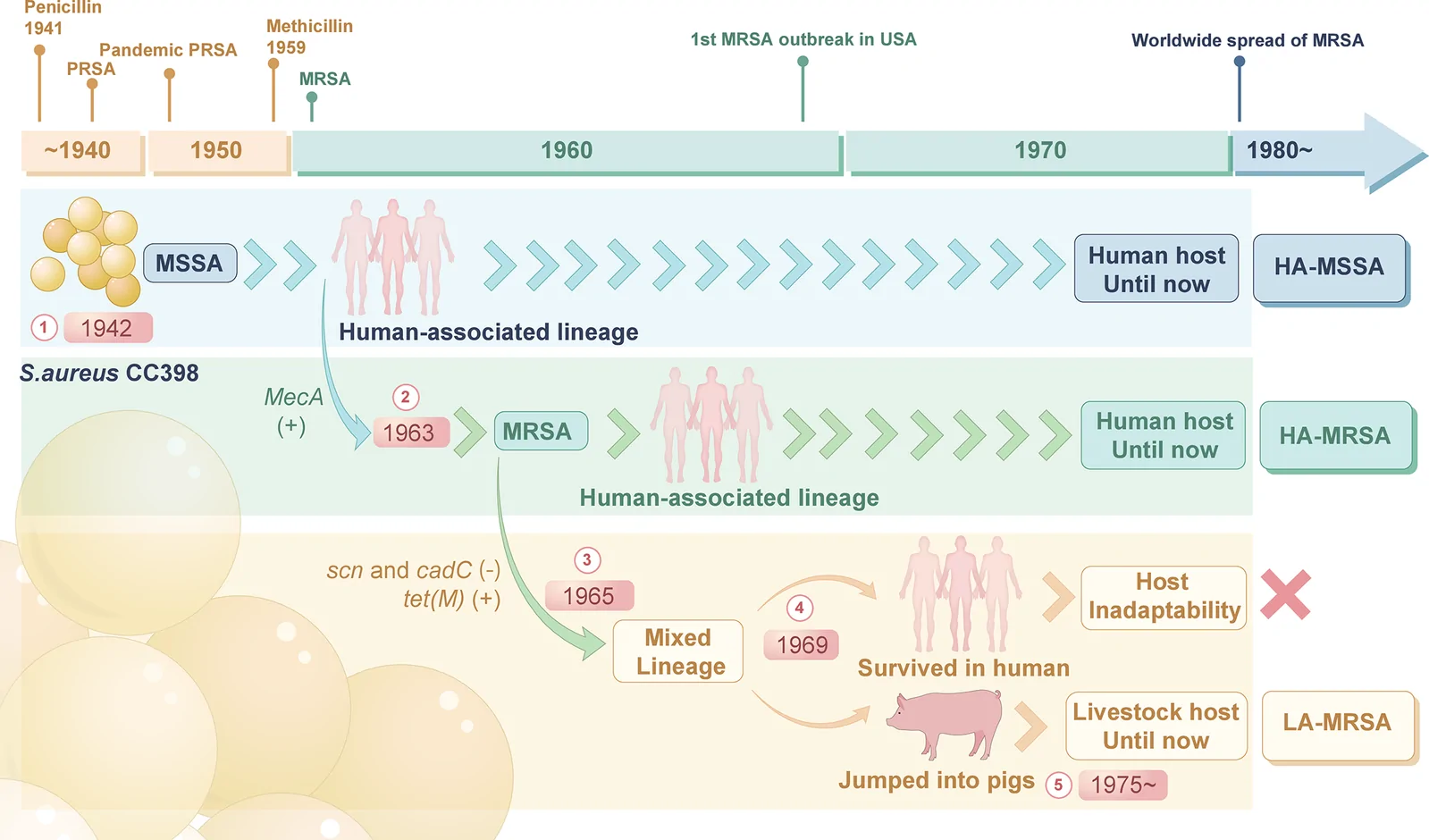
Proposed evolutionary hypothesis of *S. aureus* CC398 host adaptation. The phylogeny resolves CC398 into three major branches. The MRCA is placed at 1942 (95% CI: 1939–1945). The presence of *mecA* (~1963) and the subsequent co-occurrence of *tet*(M) with the absence of *scn* (~1965–1969) coincided with a host jump from humans to livestock. The absence of *cadC* is also temporally associated with this transition. The LA-MRSA lineage expanded after 1975, becoming globally dominant. The model is based on phylogenetic reconstruction and gene distribution patterns and has not been experimentally validated.

This study proposes an evolutionary hypothesis for *S. aureus* CC398 comprising three major branches defined by host association (Fig. 6). The time-calibrated phylogeny places the most recent common ancestor at 1942 (95% CI: 1939–1945), giving rise to the human-associated MSSA lineage. Around 1963 (95% CI: 1962-1965), the acquisition of *mecA* coincided with the emergence of HA-MRSA. Between 1965 and 1969 (95% CI: 1963–1971), the acquisition of *tet*(M) and the loss of *scn*—events well-documented in prior work—were temporally coincident with a host jump from humans to livestock (8, 29). The loss of *cadC*, a chromosomal cadmium resistance regulator identified in this study as HA-lineage-enriched, is also temporally associated with this transition, although its functional significance remains undetermined. Following this host jump, the LA-MRSA lineage expanded rapidly after 1975, becoming the dominant CC398 population globally through dissemination linked to international pig product trade, particularly fresh swine offal. The proposed evolutionary model is based on phylogenetic reconstruction together with the temporal distribution of lineage-specific genes.

## DISCUSSION

Over the past century, the global swine industry has undergone profound transformation through the expansion of international trade and intensive farming, becoming an important driver of socioeconomic development (30). Nevertheless, the interplay between animal husbandry and the evolution of microbes remains to be elucidated (31). *S. aureus* is a conditional pathogen that contaminates food, water, or facilities, leading to human illness throughout the food supply chain (32). Pigs are currently considered to be an important reservoir for livestock-associated MRSA (6, 33, 34). However, whether the global dissemination of CC398 is primarily driven by infected swine or contaminated pork products remains unclear. Likewise, the extent to which the expansion of intensive swine farming and international pork trade since the 1980s has shaped the evolution and dissemination of CC398 remains poorly understood. Here, we address these questions.

Initially, we introduced the concepts of core-genome MLST and hierarchical clustering in this study. cgMLST is a gene-by-gene approach that extends traditional MLST from seven housekeeping loci to the full complement of core genes shared across a bacterial population. A global data set encompassing 30,156 *S. aureus* isolates was organized to give a comprehensive overview of its population structure. In our high-quality species-representative data set, 293 out of 2,927 strains (10.0%) could not be classified into CCs due to the inability to match their ST types. To address the limitations of MLST, we employed HierCC, a method capable of assigning bacterial genomes to multiple levels of clusters in real time, thereby capturing a broad range of genetic diversity (19). A previous evaluation demonstrated that in *Escherichia coli*, cluster assignments at the HC1100 level could effectively distinguish ST complexes (19). Based on the results of HierCC, we defined HC650 as the optimal clustering level for *S. aureus*, as it demonstrated the highest concordance with traditional CCs. Additionally, hierarchical clustering offers the advantage of effectively determining the genetic distance between core genes of bacteria (19). Notably, the use of core genome variation represents a deliberate methodological choice rather than a simplification. While whole-genome SNP (wgSNP)-based approaches are widely used in genomic epidemiology, their application to large-scale data sets is often limited by substantial computational demands and reduced scalability. In contrast, the cgMLST framework enables standardized and computationally efficient comparisons across thousands of genomes, making it particularly suitable for global surveillance studies. From an epidemiological perspective, this method can indicate that strains associated with recent transmission events exhibit high genomic relatedness at the sequence level. Consequently, we identified HC6 as a benchmark for genomic analysis of *S. aureus* strains involved in close or covert transmission events, particularly in confirmed outbreaks. Importantly, this threshold is supported by the observed correlation between core gene allelic distances and whole-genome SNP distances, indicating that cgMLST-based metrics retain sufficient resolution to capture transmission events. Furthermore, commonly used SNP thresholds for defining recent transmission typically fall within the range of ~5–25 SNPs in previous studies (35–37), suggesting that our HC6 cutoff is broadly consistent with established epidemiological criteria while providing a more scalable framework for large-scale analyses.

Subsequently, this study identified 1,119 core genes and HC6 to assess the genomic similarity of *S. aureus* strains originating from various nations. We revealed that the CC398 lineage experienced frequent cross-border dissemination in the early 2000s, predominantly among European nations like Germany and the Netherlands, which are closely tied to the livestock trade. This finding is consistent with the fact that both Germany and the Netherlands are leading exporters in the livestock industry (38, 39). Moreover, the persistence of transnational clusters over multiple years suggests the existence of a persistent reservoir of CC398 within international livestock trade networks. Swine are likely to serve as the primary reservoir. Intensive pig farming, which became widespread during the late 20th century, increased pig-to-pig contact while reducing pig-to-human contact (40). Such farming practices may facilitate the transmission and long-term persistence of CC398 within swine populations.

A key finding of this study is the distinct evolutionary trajectory of CC398 in East Asia. Fernandez et al. established the global evolutionary timeline; our analysis extends that framework by resolving several previously undescribed East Asian phylogroups (9). SAP1, the most notable, is a multidrug-resistant, multi-host clade that lacks the immune evasion cluster genes and was prevalent in China between 2010 and 2019 before declining. SAP2, which retains *sak* but lacks *scn*, may represent spillover infections from environmental or animal sources rather than sustained human-to-human transmission (41).

Additionally, this study revealed SAP3 as a distinctive MRSA lineage, exclusively originating from Taiwan and forming a unique cluster primarily associated with infections in Taiwan (41). This suggested a localized evolutionary trajectory, warranting further bacterial isolation and monitoring to better understand this cluster. Meanwhile, AP2 is a human MRSA lineage that carries the *chp-sak-scn* gene cluster, indicating its potential role as one of the primary clusters responsible for human clinical infections in Asia.

Furthermore, this study observed differences in gene copy rates between human host strains in the HA and LA lineages. Specifically, the gene copy rates of human host strains in the LA lineage were more similar to those of animal host strains within the same lineage, differing from those of human host strains in the HA lineage. The AAP, EP3, EP4, and EP5 phylogenetic groups had shown an increasing trend in China in recent years. Pigs emerged as the only host capable of spreading to all other hosts, underscoring their critical role in host jump events. Despite the Chinese LA-CC398 lineage constituting only 23% of the entire phylogeny, pig hosts played a pivotal role in facilitating these transmission dynamics.

Previous studies have systematically investigated the antimicrobial resistance and virulence profiles of *S. aureus* CC398 across multiple countries using time-calibrated phylogenetic analyses (41). Their study elucidated the evolutionary timeline and transmission routes of strains from various regions of Eurasia, particularly highlighting the two important European lineages, EP4 and EP5. Extending this work, our research expands on these findings by quantifying the geographic distribution and comparing the strain distributions of HA and LA lineages across countries. In European pig-producing countries, the prevailing strains belonged to the LA lineage and harbored a higher number of antimicrobial resistance genes, which likely reflects the extensive application of antimicrobials within intensive farming systems. Conversely, the MRSA and MSSA strains isolated in China are mainly associated with the HA lineage, exhibiting a unique pattern of strain distribution and AMR profiles.

Another highlight of this project is the introduction of the Harvard Trade Database as a reference. In Fig. 5D, each point represents a country-to-country pair, comparing LA-CC398 transmission events with pork offal trade volumes between the same two countries. Based on correlation analysis, we revealed a significant association between global pig trade and the spread of LA-CC398, indicating that the dominance of modern pig industries in Europe has made them a central hub for the development and global dissemination of *S. aureus*. Such CC398, widely distributed in pig farms, could spread through human-to-human contact, cutting tools, and any surface directly exposed to pork during handling and processing, underscoring the role of intensive farming in driving the geographic spread of foodborne pathogens (42). However, these observations are hypothesis-generating only, and further field-based epidemiological investigations are needed to establish any causal relationship.

Furthermore, among the various pig-derived products, fresh pork offal stands out as the primary vehicle for pathogen transmission. There is evidence suggesting that the extensive use of offal in feeding practices can lead to increased secondary transmission of pathogens, which has been documented with related pathogens, including Salmonella, African swine fever virus, and foot-and-mouth disease virus (43–45). Significant attention should be paid to the storage conditions of pork products, with an emphasis on fresh offal. Non-freezing storage techniques may inadvertently serve as a medium for bacterial survival and dissemination, potentially facilitating the spread of livestock-enriched pathogens.

The evolutionary trajectory proposed here is consistent with earlier work showing that CC398 originated as a human-associated MSSA lineage and that the acquisition of *tet*(M) and loss of the IEC genes marked the transition to the LA lineage (8, 29). This study adds the loss of *cadC*—a chromosomal cadmium resistance regulator—as a novel temporal association with this host jump. Unlike *tet*(M) and *scn*, *cadC* remains unvalidated; whether its loss contributed causally to the host jump or was a passenger event driven by relaxed selection for cadmium resistance in the livestock environment requires experimental investigation. Following the host jump, the LA-MRSA lineage expanded globally through industrial pig trade after 1975, giving rise to the dominant LA-CC398 population observed today.

This study has three principal limitations. First, the species-representative data set overrepresents the United States (28% of isolates), and pre-2000 sampling is sparse. Year-stratified down-sampling and country-level exclusion analyses confirmed that the observed temporal trends and cluster rankings are robust to these imbalances; nevertheless, the apparent prevalence of clones such as CC5 and CC8 may be partly inflated, and pre-2000 inference remains constrained. Second, the CC398 phylogeny is enriched for European isolates, and transition counts correlate with sampling depth (R = 0.80, *P* < 0.0001). Normalization confirmed that the core transmission patterns—Swine-to-Human predominance, the European hub role, and limited outward transmission from China—are robust, although specific country-to-country routes should be interpreted with caution. Third, the genetic diversity of CC398 in developing regions, particularly Africa, remains largely unsampled; absence from these regions does not constitute evidence of true absence.

### Conclusion

This study documents a shift in the global population structure of *S. aureus*, with CC398 emerging as a dominant clone after the 2000s. By applying a novel cgMLST scheme to over 30,000 genomes, we show that CC398 undergoes frequent cross-border dissemination, with transmission networks closely aligned to international trade in fresh pork products—particularly offal and fresh meat. Pigs serve as the predominant reservoir driving this global expansion, accounting for the majority of host jump events. However, Chinese CC398 follows a markedly different trajectory: it remains a persistent human-associated MSSA lineage forming unique East Asia-specific phylogroups, with LA-MRSA appearing only after 2019 as mixed multinational clusters. These findings challenge the prevailing model of uniform CC398 dissemination and highlight how regional contexts—including livestock practices and trade networks—produce distinct evolutionary outcomes. The integration of large-scale genomics with trade data provides a framework for regionally tailored MRSA surveillance within a One Health approach.

## Data Availability

All newly sequenced genomes have been deposited in NCBI under BioProject accession numbers PRJNA1190906, PRJNA1048050, and PRJNA1191548. Individual accession numbers for the 898 newly sequenced isolates are listed in Table S20. Public genome accessions and associated metadata are provided in Table S1 (*n* = 30,719), with the quality-filtered representative subset in Table S2 (*n* = 2,927). The CC398 lineage data set (*n* = 3,642) and outbreak analysis data set (*n* = 358) are detailed in Tables S8 tand S11, respectively. The cgMLST scheme and analysis code have been uploaded to GitHub (https://github.com/Guilai-Jiang/StaphMix) and are available for download at https://pathobase.iotabiome.com/.

## SUPPLEMENTAL MATERIAL

Supplemental figures part 1 msystems.00621-26-s0001.pdf Fig. S1 to S6.

Supplemental figures part 2 msystems.00621-26-s0002.pdf Fig. S7 to S10.

Supplemental figures part 3 msystems.00621-26-s0003.pdf Fig. S11 to S14.

Supplemental Tables msystems.00621-26-s0004.xlsx Tables S1 to S20.

ASM does not own the copyrights to Supplemental Material that may be linked to, or accessed through, an article. The authors have granted ASM a non-exclusive, world-wide license to publish the Supplemental Material files. Please contact the corresponding author directly for reuse.
