# Supplementary material for: Large-scale genomic analysis places Chinese CC398 as a persistent human-associated MSSA lineage apart from the dominant global LA-MRSA clade
Source: mSystems. 2026 Sep 11;11(10):e00621-26. doi: 10.1128/msystems.00621-26 (PMC13713001; doi:10.1128/msystems.00621-26)

Supplementary Figure 1

Pan-genome rarefaction curve for the representative dataset of the species. As the number of strains increases incrementally (x-axis), the numbers of core and pan genes (y-axis) are recorded. Strains are added in a random order, with 10 replicate experiments conducted. Differences are shown as shaded areas, representing the 95% confidence interval.

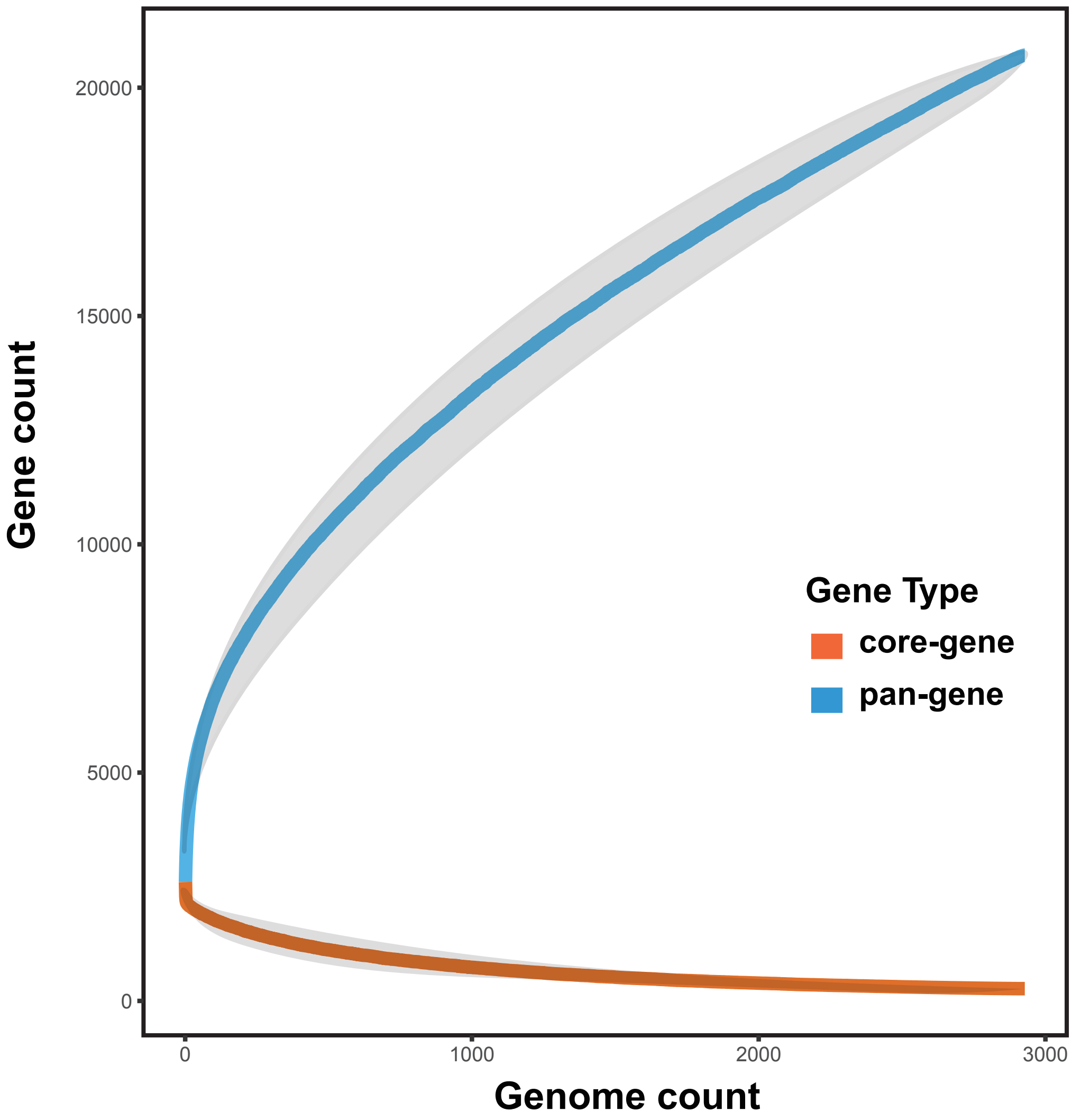

Supplementary Figure 2

Evaluation of two published cgMLST schemes using EToKi. "Initial core genes" indicate the number of core genes detected in the species-representative dataset. The Venn diagrams summarize genes excluded because of low conservation (presence < 0.95), low completeness (completeness < 0.94), or high allelic variability (sigma > 3). Each circle represents one filtering criterion, and overlapping regions indicate genes that failed multiple criteria simultaneously. (Left) 2,208-gene PubMLST cgMLST scheme. (Right) 1,861-gene SeqSphere+ cgMLST scheme.

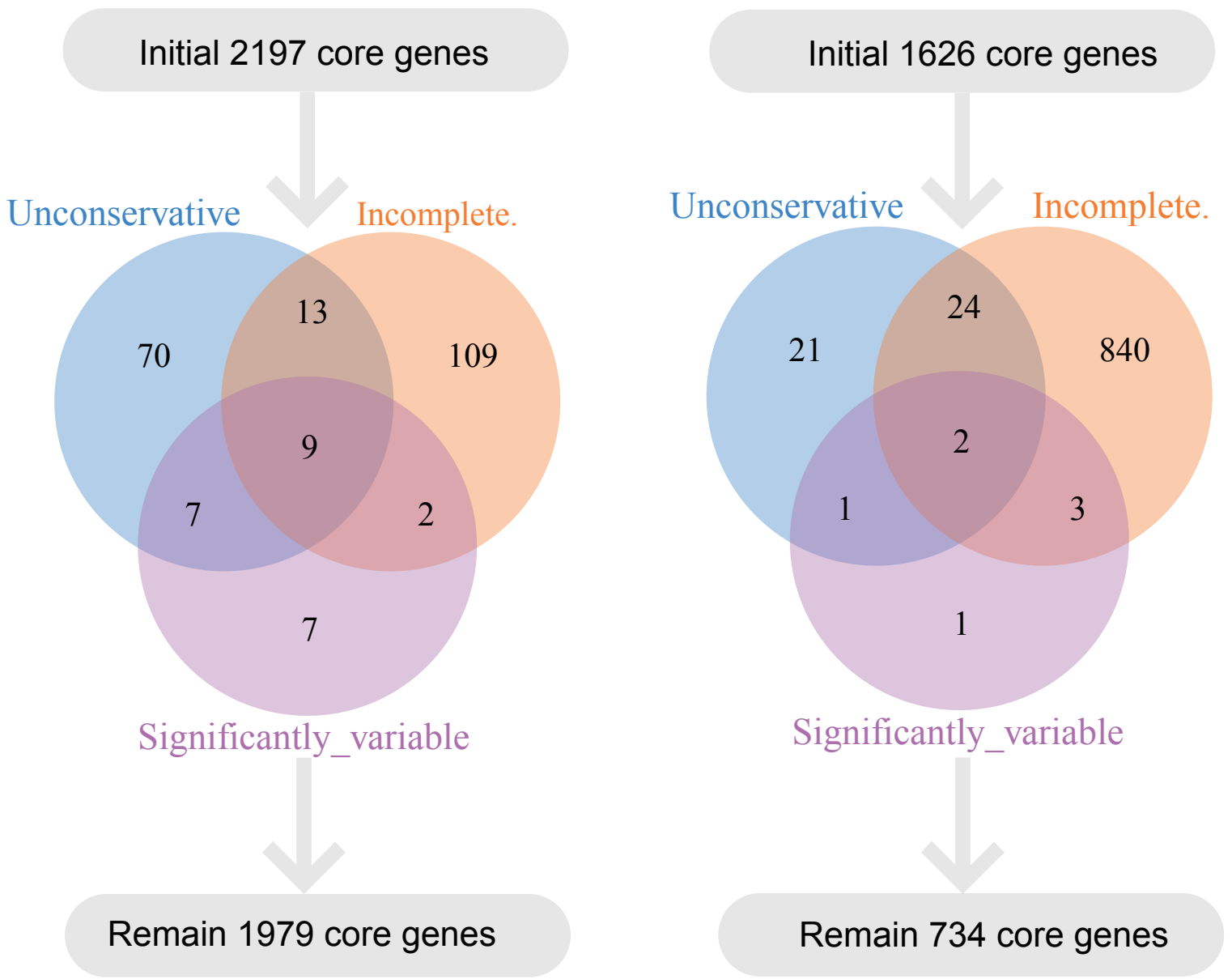

Supplementary Figure 3

Distance between paired alleles in the representative dataset and silhouette coefficient scores for assessing the quality of different HC clusters.

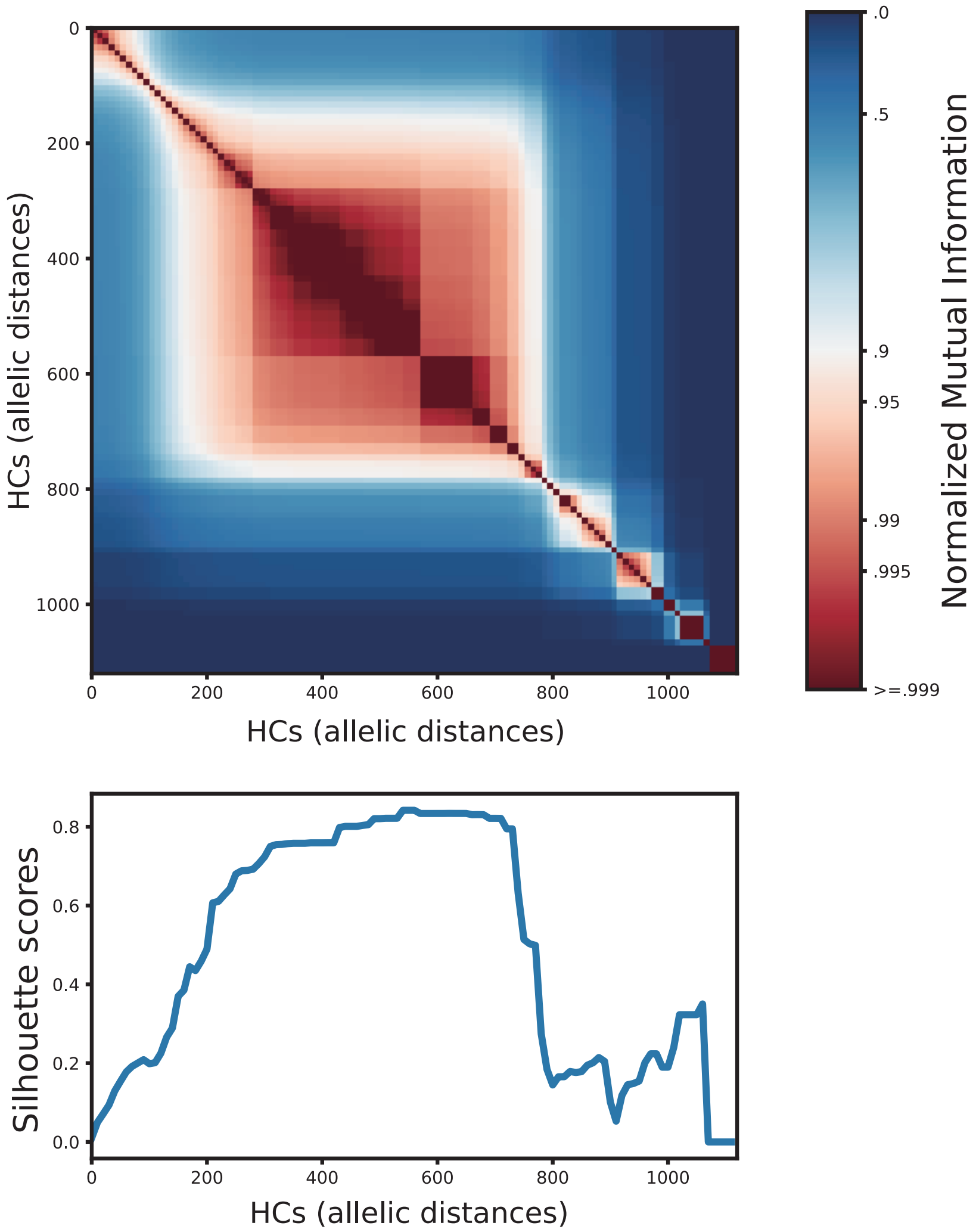

Supplementary Figure 4

Sensitivity of HC650 cluster temporal composition to year-stratified down-sampling. Original versus down-sampled proportions of the seven most abundant HC650 clusters across 5-year bins. Original proportions (circles, solid line) were computed from all available isolates in each bin. Down-sampled proportions (squares, dashed line) represent the mean of 1,000 bootstrap iterations after randomly down-sampling each bin with >100 strains to a cap of n = 100. Shaded areas indicate the 95% confidence interval. All original proportions fall within the 95% CI.

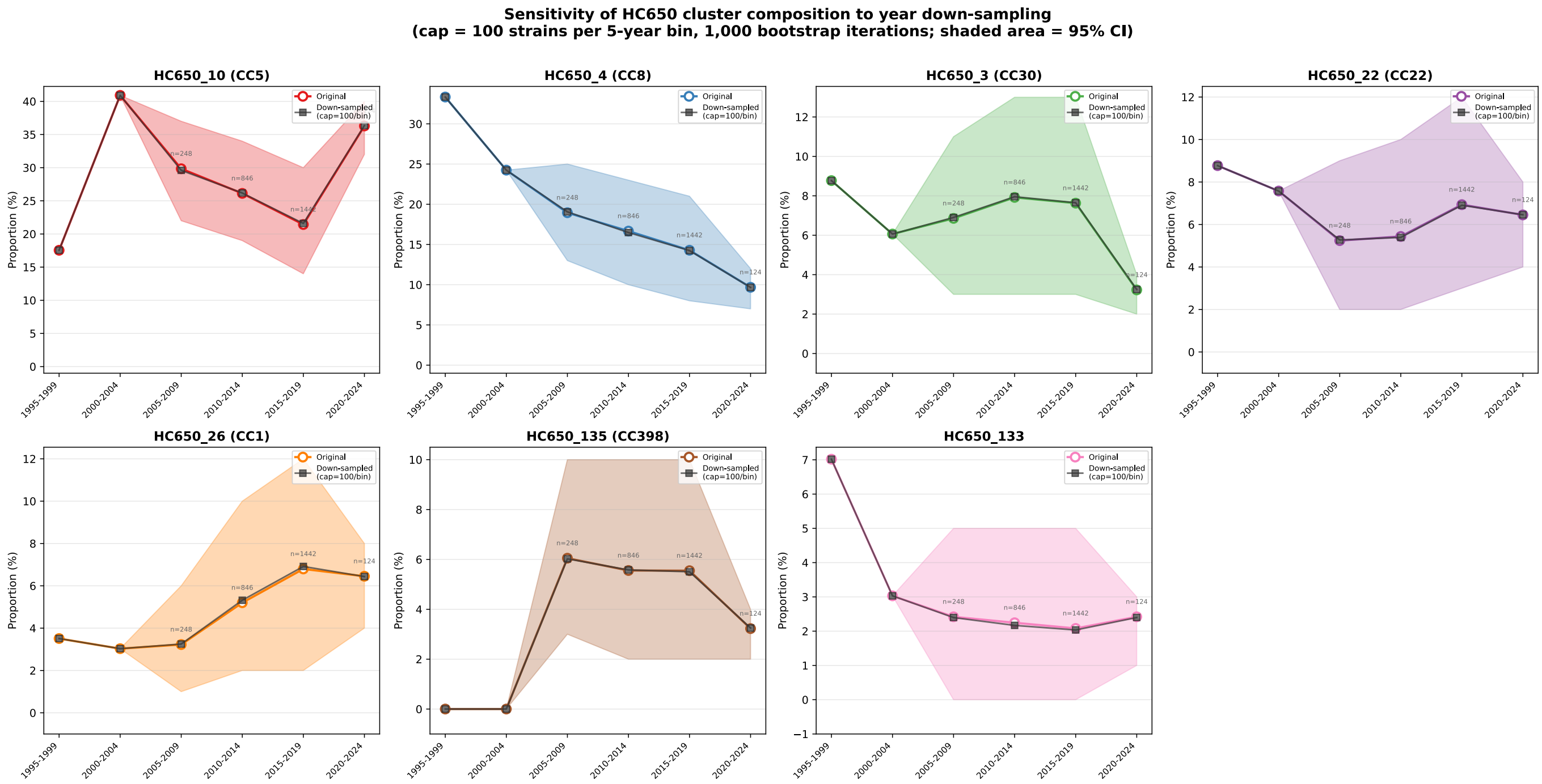

Supplementary Figure 5

Sensitivity of S. aureus population structure to geographic sampling. (A) Leave-one-country-out analysis. Heatmap showing the change in HC650 cluster proportions (percentage points) after excluding each of the ten most-represented countries. Blue, decrease; red, increase. (B) Country-stratified bootstrap resampling (1,000 iterations). Violin plots show the bootstrap distribution of HC650 cluster proportions; black diamonds indicate observed full-dataset values.

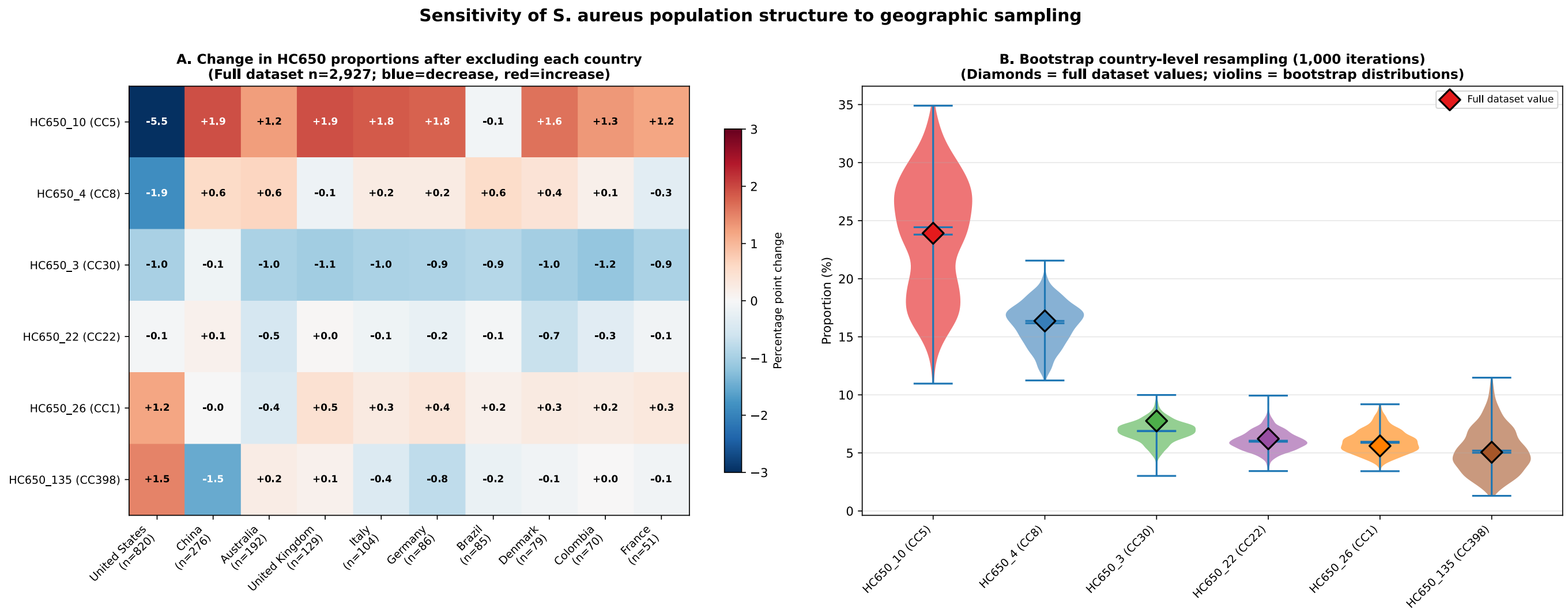

Supplementary Figure 6

Effect of HC6-level clonal dereplication on AMR gene distributions. (A) Stability of country-level AMR gene rankings after HC6 dereplication. Horizontal bars show the Spearman rank correlation (rho) between pre- and post-dereplication country means for all 31 AMR genes. The dashed red line indicates the median rho (0.988). All correlations are significant (P < 0.001). (B) Global mean copy number of the 12 AMR genes with the highest Spearman rho, before (red) and after (blue) HC6 dereplication.

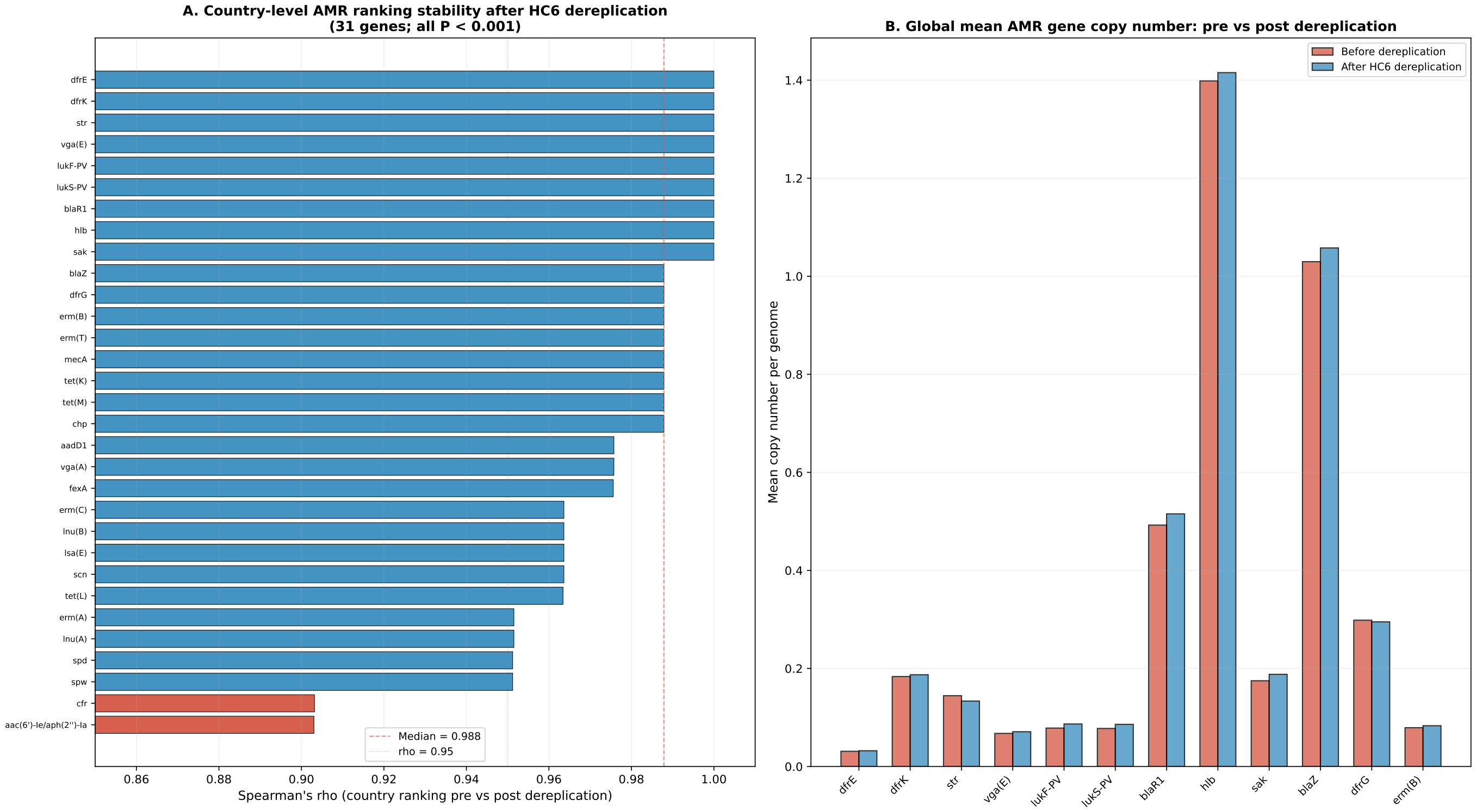

Supplement: Supplementary file 1 [file msystems.00621-26-s0001.pdf]
