# Supplementary material for: Large-scale genomic analysis places Chinese CC398 as a persistent human-associated MSSA lineage apart from the dominant global LA-MRSA clade
Source: mSystems. 2026 Sep 11;11(10):e00621-26. doi: 10.1128/msystems.00621-26 (PMC13713001; doi:10.1128/msystems.00621-26)

Supplementary Figure 7

Receiver Operating Characteristic (ROC) curve for the Random Forest model. The blue curve represents the performance of the Random Forest model in distinguishing between HA and LA lineages, with an area under the curve (AUC) of 0.9991. The red dashed line indicates the performance of a random classifier (AUC = 0.5).

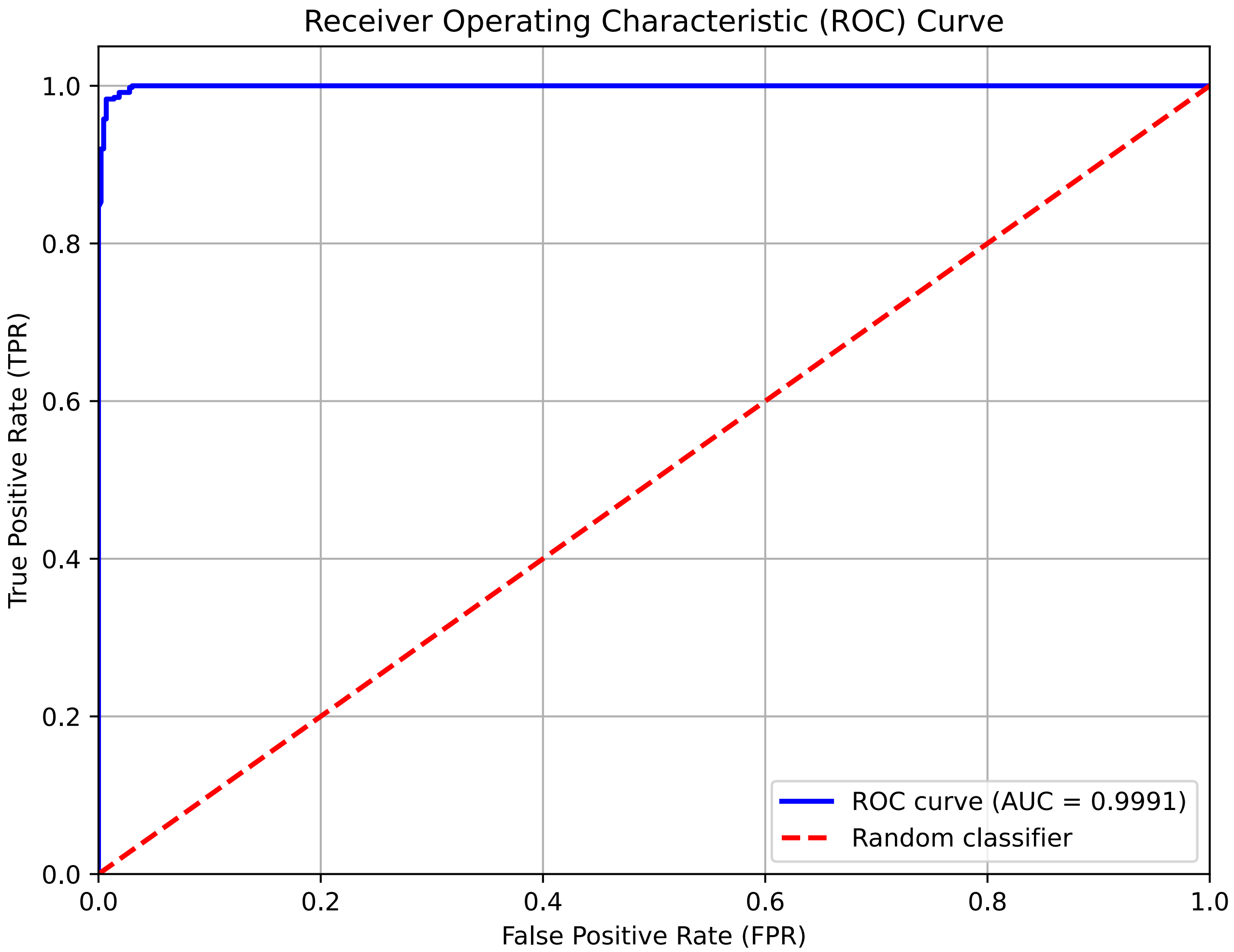

Supplementary Figure 8

Within-lineage host-stratified analysis of cadC. (A) cadC presence in four groups defined by lineage and host. cadC is enriched in HA-lineage human isolates (75.4%) versus animal isolates (37.8%; OR = 5.05, P < 0.0001). (B) cadC copy number distribution by lineage and host. (C) Mean gene copy numbers for cadC versus the average of HA- and LA-lineage-enriched genes identified by Random Forest.

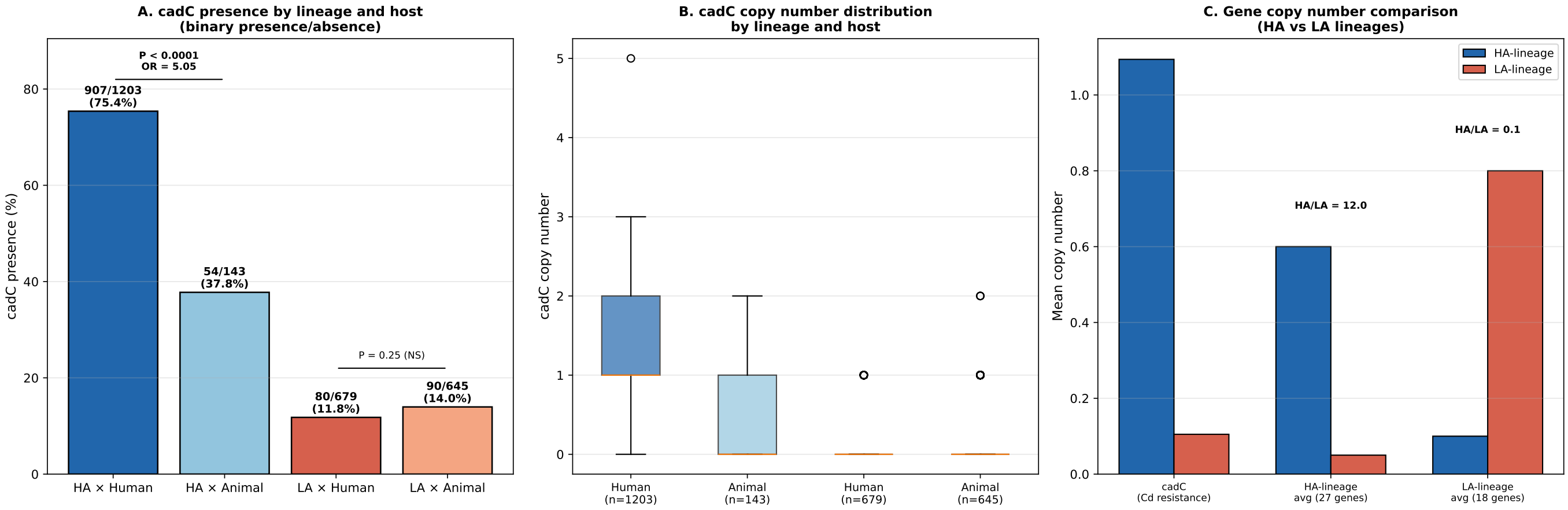

Supplementary Figure 9

cadC prevalence by country within the HA lineage. cadC presence (blue bars) and mean copy number (red bars) in the 12 countries with the largest HA-lineage CC398 populations (n >= 10). The consistent enrichment across diverse geographic regions supports cadC as a lineage-associated feature.

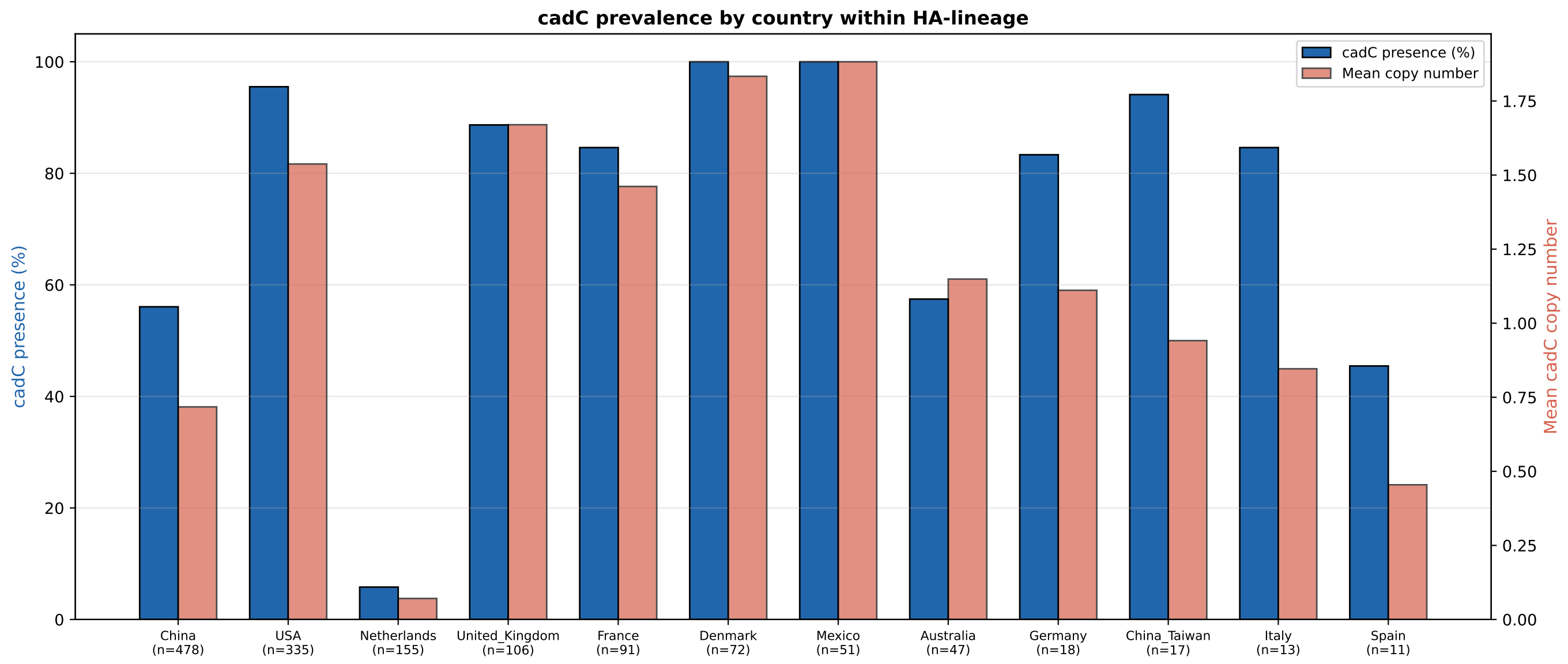

Supplementary Figure 10

Leave-one-clade-out (LOCO) validation of cadC. (A) RF accuracy when each CC398 subclade is held out. n values indicate the number of strains in the held-out clade. (B) cadC feature importance rank in each LOCO fold. (C) cadC prevalence by subclade. Blue bars, HA-lineage clades; red bars, LA-lineage clades.

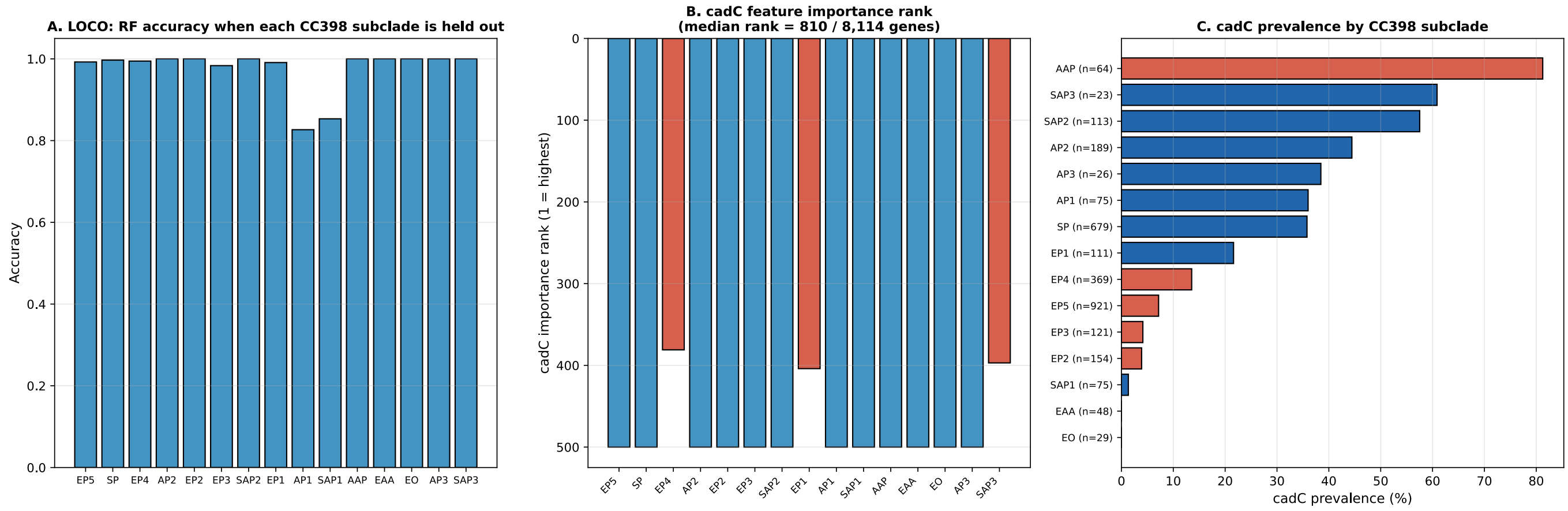

Supplement: Supplementary file 2 [file msystems.00621-26-s0002.pdf]
