# Supplementary material for: Large-scale genomic analysis places Chinese CC398 as a persistent human-associated MSSA lineage apart from the dominant global LA-MRSA clade
Source: mSystems. 2026 Sep 11;11(10):e00621-26. doi: 10.1128/msystems.00621-26 (PMC13713001; doi:10.1128/msystems.00621-26)

Supplementary Figure 11

Host transition matrix and per-host transition balance in CC398. (A) Heatmap of the host-to-host transition matrix inferred from ancestral state reconstruction on the CC398 time-calibrated phylogenetic tree. Rows represent source hosts, columns represent target hosts, and cell values are raw transition counts. (B) Incoming and outgoing transition counts per host category. Blue bars indicate incoming transitions; red bars indicate outgoing transitions. The number of tips (n) in each host category is annotated above the bars.

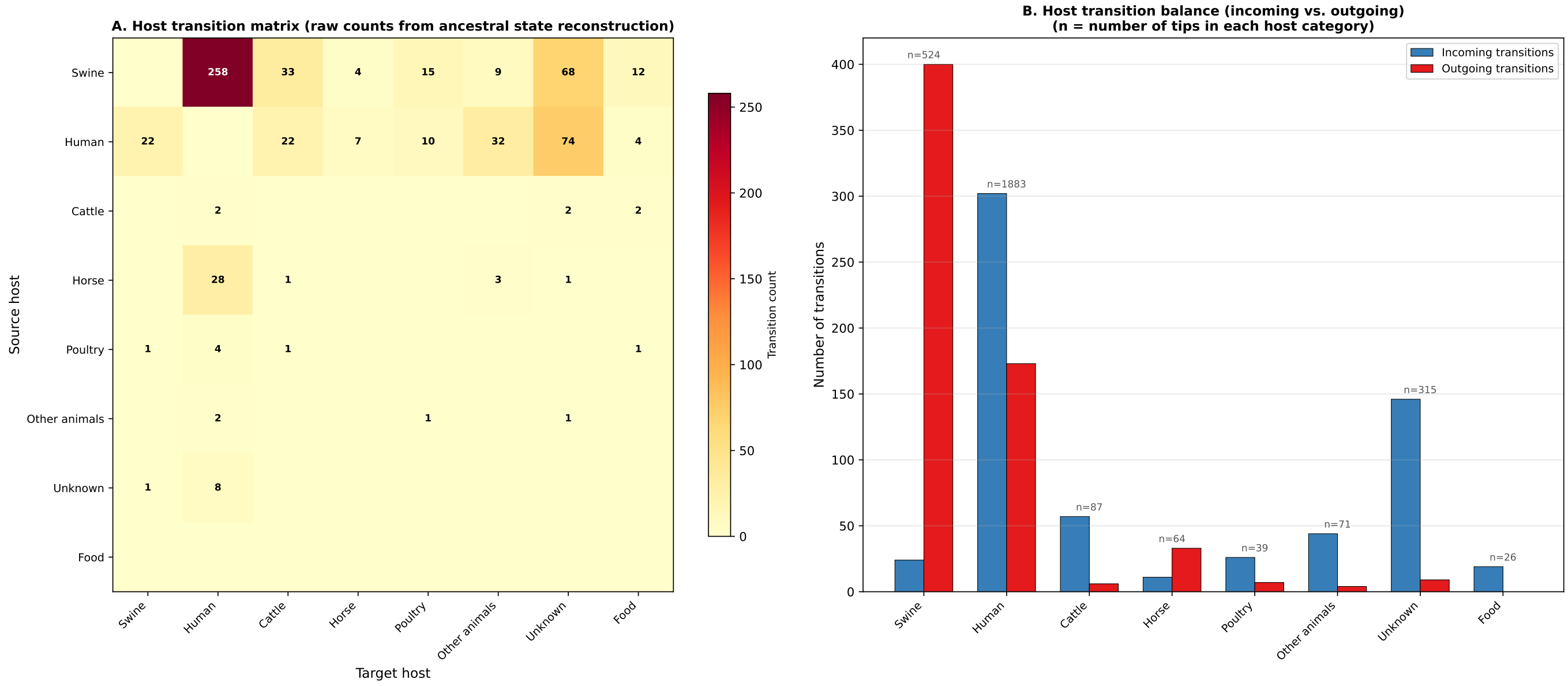

Supplementary Figure 12

Relationship between country sampling depth and inferred transition counts. (A) Per-country sampling depth versus the number of outgoing transitions inferred from ancestral state reconstruction. The dashed line shows the power-law fit. (B) Per-country sampling depth versus incoming transitions. Countries with >=50 tips or >=20 transitions are labelled. Pearson correlation coefficients and P values are annotated.

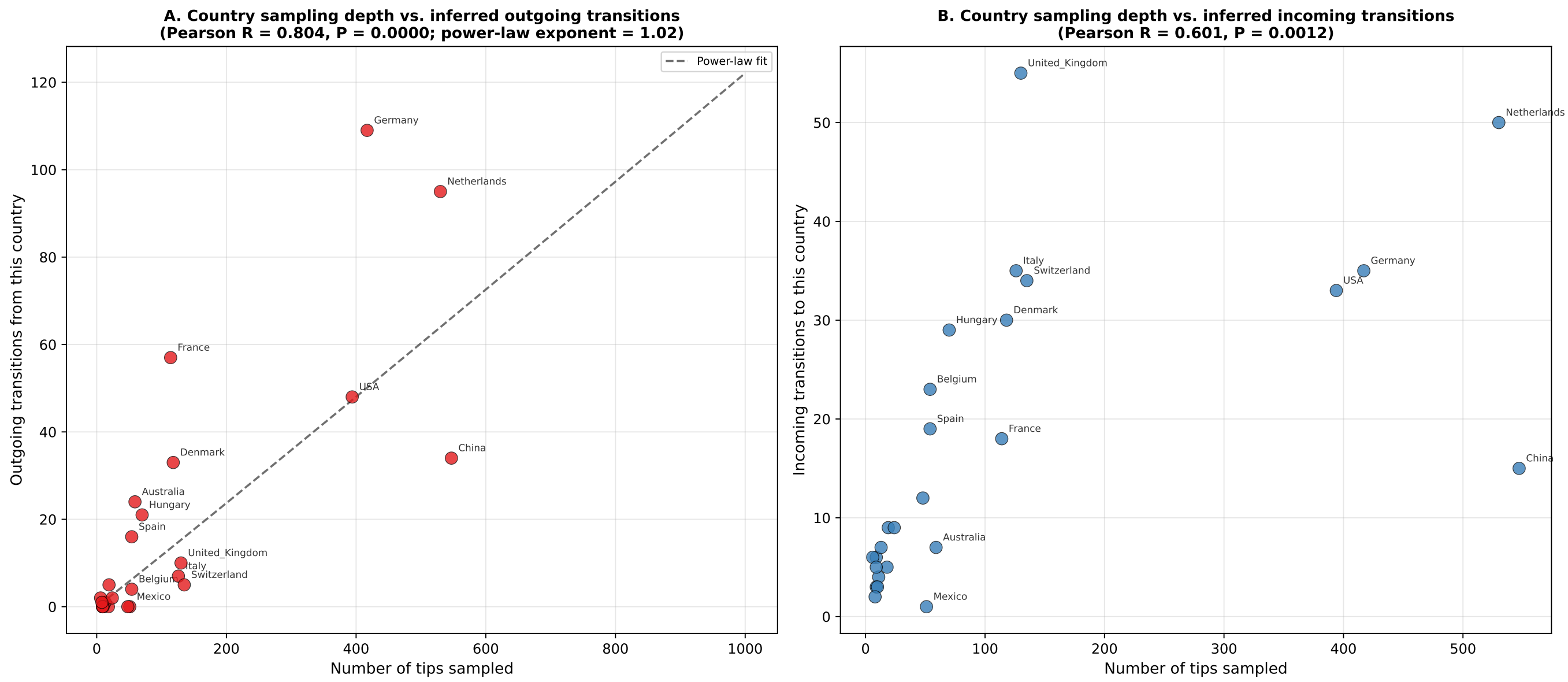

Supplementary Figure 13

Comparison of raw and sampling-normalized country-to-country transition counts. (A) Top 20 country-to-country transition pairs ranked by raw count. (B) Top 20 pairs ranked after normalizing each transition count by the geometric mean of source and target country tip counts. Numbers in parentheses indicate tip counts per country. Normalization reduces the dominance of heavily sampled countries while preserving the overall transmission structure.

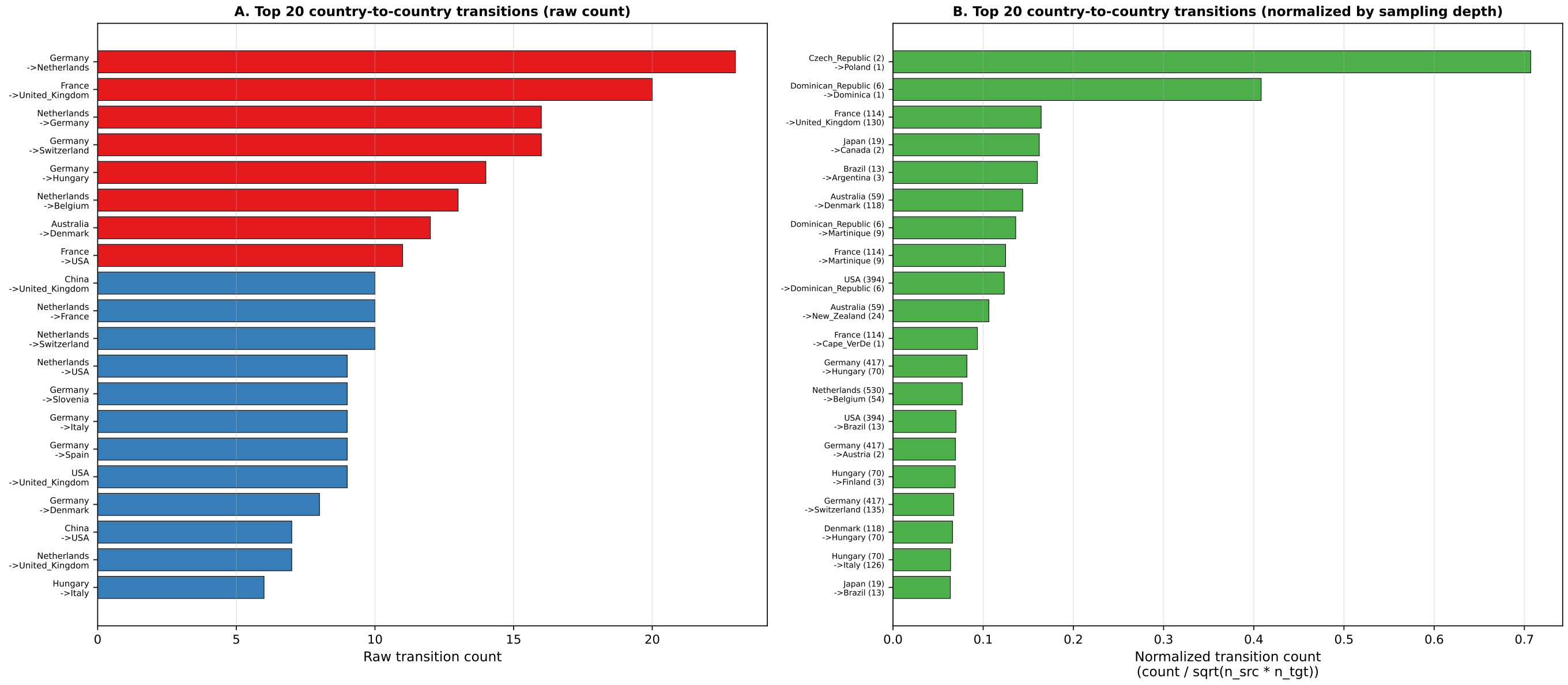

Supplementary Figure 14

Per-capita transition rates by country. (A) Per-country transition involvement versus sampling depth. Bubble size and colour represent transitions per tip. The dashed line indicates the expected relationship under proportionality to sampling. (B) Transitions per tip for the twelve most-sampled countries, stratified by direction (blue, incoming; red, outgoing). n values indicate the total number of CC398 tips per country.

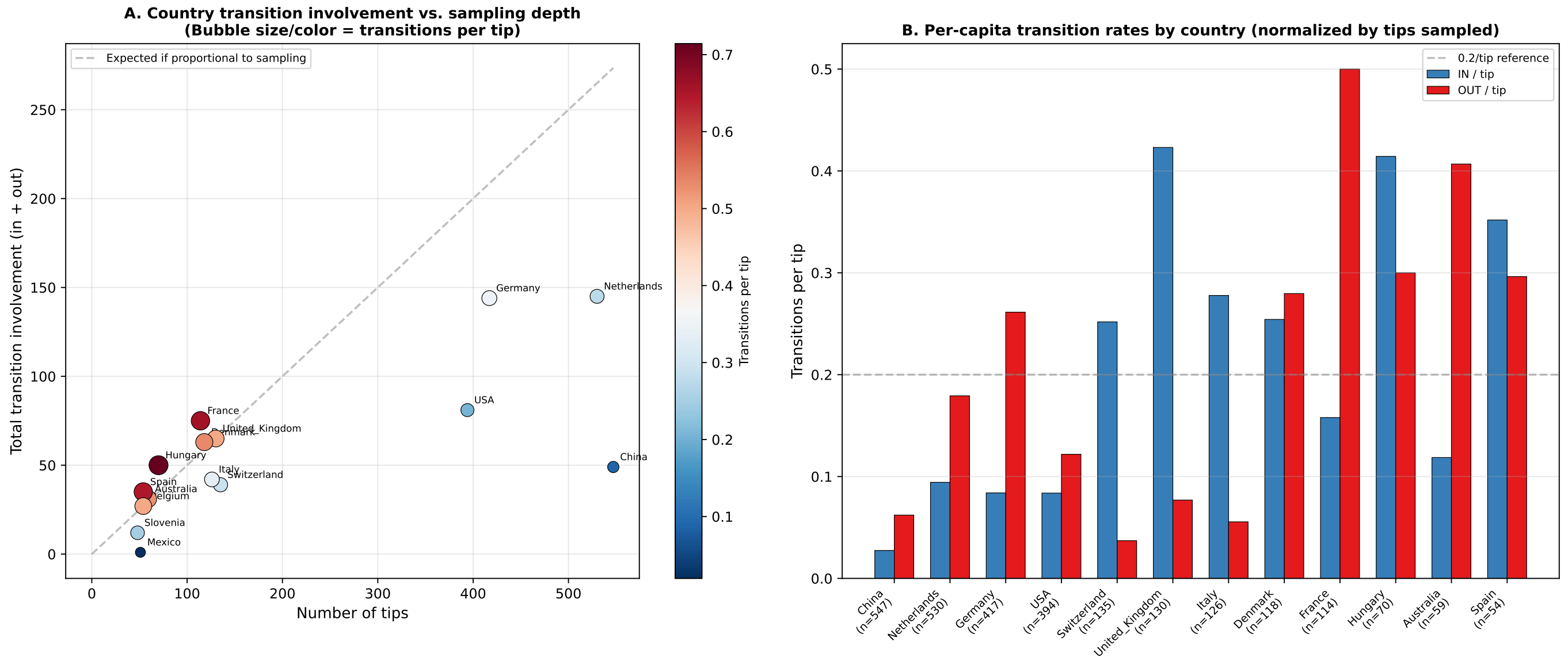

Supplement: Supplementary file 3 [file msystems.00621-26-s0003.pdf]
